# A new tiny toad species of *Amazophrynella* (Anura: Bufonidae) from east of the Guiana Shield in Amazonia, Brazil

**DOI:** 10.7717/peerj.9887

**Published:** 2020-09-18

**Authors:** Sarah Mângia, Ricardo Koroiva, Diego José Santana

**Affiliations:** 1 Instituto de Biociências, Universidade Federal de Mato Grosso do Sul, Campo Grande, Mato Grosso do Sul, Brazil; 2 Departamento de Sistemática e Ecologia, Universidade Federal da Paraíba, João Pessoa, Paraíba, Brazil

**Keywords:** Amazon Forest, Brazil, Conservation, Systematic, Taxonomy

## Abstract

The combination of different approaches has successfully delimited new species within many Neotropical species complexes traditionally classified as a single nominal organism. Recent studies have shown that the Amazonian endemic genus *Amazophrynella*, currently composed of 12 small-sized species, could harbor several additional species. Based on morphology and molecular data, we describe a new species of *Amazophrynella* from east of the Guiana Shield, in Pará state, Brazil. The new species is characterized by having one of the biggest size of the genus (SVL of males 16.0–17.8 mm and females 22.9–24.4 mm), presence of a large palmar tubercle (occupying 2/4 of the palmar surface), 5.6–8.1% uncorrected *p*-distance from its sister clade (including *A. teko*, *A.* sp.1, and *A. manaos*) for the 16S mitochondrial gene, and 8.8% for the COI. The new species described here represents a newly discovered lineage. Of the 12 *Amazophrynella* species currently recognized, two were describe in the last century (*A. bokermanni* and *A. minuta*) and the remaining species were recently discovered and described (in the last six years), which underscores the degree to which species richness of *Amazophrynella* is underestimated.

## Introduction

Combining different approaches (e.g., morphological, bioacoustic, population genetics, ecology) have been successfully used to delimit new species in the Neotropics (e.g., Andrade et al., 2019; Mângia et al., 2018; Pereira et al., 2018), and revealed new taxa within many species complexes traditionally classified as a single nominal organism (Fouquet et al., 2012a; Fouquet et al., 2014; Gehara et al., 2013). Although the megadiverse Amazon Forest biome harbors many species that are widespread, many anurans are philopatric with poor dispersal abilities, which frequently results in strong genetic structure and allopatric distributions (Funk, Caminer & Ron, 2012; Pirani et al., 2020; Vacher et al., 2017). Such patterns have been observed in closely related species that were traditionally considered a single one, widely distributed in the Amazon (Ferrão et al., 2016; Maia, Lima & Kaefer, 2017).

The Amazonian genus *Amazophrynella* Fouquet, Recoder, Teixeira, Cassimiro, Amaro, Camacho, Damasceno, Carnaval, Moritz and Rodrigues, 2012 was created to allocate two species (*A. bokermanni* and *A. minuta*) previously placed in *Dendrophryniscus* Jiménez de la Espada, 1870. The genus was recognized as a morphologic and genetically lineage deeply divergent from the Atlantic Forest genus, *Dendrophryniscus* (Fouquet et al., 2012b). Rojas et al. (2014) described *Amazophrynella manaos* from the Guiana Shield region previously identified as *A. minuta* based on morphological and molecular evidences. Afterword, Rojas et al. (2018a) sampled many populations and reviewed the morphology and acoustic variation within the genus, and discussed its molecular phylogeny, naming several new species and a considerable number of other candidate species. Currently, species of *Amazophrynella* occur in Venezuela, Brazil, Bolivia, Peru, Ecuador, Colombia, Guyana and French Guyana, inhabiting primary forest leaf litter (Frost, 2020; Rojas et al., 2015).

Nowadays, the genus contains 12 described species, of which 10 have been described in the last six years, all recovered as genetically distinct lineages (see Kaefer et al., 2019). Despite the high number of descriptions, recent studies have shown that species richness of this genus is underestimated, and several undescribed species likely exist (Fouquet et al., 2012a; Rojas et al., 2016; Rojas et al., 2018a; Vacher et al., 2020). Emphasizing the existence of many species still unknown for the genus, we collected a series of small-sized bufonid specimens in Óbidos municipality, Pará State, Brazil, that represents a genetically structured lineage within the genus *Amazophrynella*, which we describe as a new species.

## Materials & Methods

### Sampling

We conducted visual surveys, and used pitfall traps as a complementary method, at Óbidos municipality, Pará state, Brazil in January-February 2015. All specimens were captured manually and killed using 5% lidocaine, fixed in 10% formalin, and transferred to 70% ethanol for permanent storage (following Conselho Federal de Biologia-CFBio No 148/2012, 2012). Voucher specimens are housed in the Coleção Zoológica da Universidade Federal de Mato Grosso do Sul (acronym ZUFMS-AMP) (Appendix), Campo Grande, Brazil. We state here that appropriate protocols for the collection and handling of the individuals were followed for the present research according to Brazilian federal law. Collect permit was issue by ICMBio (SISBio 45889-1).

### Morphology

We followed Kok & Kalamandeen (2008) in taking 14 measurements of eight adult specimens (four males, four females), using a digital caliper (0.01 mm): snout-vent length (SVL), from the tip of the snout to the posterior margin of the vent; head length (HL), from the posterior edge of the jaw to the tip of the snout; head width (HW), the greatest width of the head, at the level of the posterior edges of the tympanum; eye diameter (ED)—measured horizontally across eye; internarial distance (IND), the distance between the internal edges of the nares; snout length (SL), from the anterior edge of the eye to the tip of the snout; hand length (HAL), from the proximal edge of the palmar tubercle to the tip of finger III; upper arm length (UAL), from the edge of the arm insertion to the tip of the elbow; size of finger I (FI) and finger II (FII)—measured from the finger insertion to the tip of the finger; thigh length (THL), from the vent to the posterior edge of the knee; tibia length (TL), from the outer edge of the knee to the tip of the heel; tarsal length (TAL), from the heel to the proximal edge of the inner metatarsal tubercle; foot length (FL) from the proximal edge of the inner metatarsal tubercle to the tip of toe IV (Rojas et al., 2018b). All measures were taken on the left side of the specimens, except for one male individual (ZUFMS-AMP12824) that is missing the left tibia, tarsus and foot. In this case we measured the right side.

For morphological analysis, including diagnostic characters, we observed the following characters: dorsal skin texture, ventral skin texture, head shape, shape of palmar tubercle, relative length of fingers and ventral coloration. We determined the sex of individuals using the sexual dimorphism observed upon collection of amplected couples and checking the gonads by a ventral incision (Rojas et al., 2018b). Specimens examined are listed in Appendix.

### Phylogenetic inference and genetic distances

We extracted whole genomic DNA from four specimens (ZUFMS-AMP12824, ZUFMS-AMP12821, ZUFMS-AMP12825, and ZUFMS-AMP12829) using the QIAGEN DNeasy Blood and Tissue Kit (QIAGEN). We used polymerase chain reaction (PCR) to amplify fragments of the 12S and 16S ribosomal RNA mitochondrial genes and the Cytochrome oxidase I mitochondrial gene (COI), using the primers 12SMVZ59 (5′- ATA GCA CTG AAA AYG CTD AGA TG -3′) and 12SMVZ50 (5′- TYT CGG TGT AAG YGA RAK GCT T -3′) of Graybeal (1997), 16Sa (5′-CGC CTG TTT ATC AAA AAC AT-3′) and 16Sb (5′-CCG GTC TGA ACT CAG ATC ACG T-3′) of Palumbi et al. (2002) and T3-AnF1 (’5- ATT AAC CCT CAC TAA AGA CHA AYC AYA AAG AYA TYG G-3′) and T7-AnR1 (’5- AAT ACG ACT CAC TAT AGC CRA ARA ATC ARA ADA RRT GTT G-3′) of Lyra, Haddad & Azeredo-Espin (2017), respectively. Conditions for PCR amplification consisted of 1 × buffer, dNTP at 0.2 mM, each primer at 0.2 µM, MgCl_2_ at 2mM, 1U Taq polymerase and 2 µl of template DNA, in a total reaction volume of 25 µl. The PCR cycling program was run according to Lyra, Haddad & Azeredo-Espin (2017; COI) and Andrade et al. (2019; 16s and 12s). We purified PCR products by Ethanol/ Sodium Acetate and bidirectionally sequenced them using an ABI 3130 Genetic Analyzer (Applied Biosystems).

Only one individual (the holotype ZUFMS-AMP12821) had the three genes successfully sequenced. For the other three individuals, only the COI was sequenced (see Table S1). We used GENEIOUS (v 9.0.5, Newark, NJ) (Kearse et al., 2012) to check sequence quality, edit chromatograms, and assemble contigs. Our DNA sequences were compared to and evaluated together with mtDNA fragments of 12S, 16S and COI from species of *Amazophrynella* obtained from GenBank. For overview of all samples and GenBank accession numbers see Table S1. We aligned sequences for each gene loci using Muscle v3.8.425 (Edgar, 2004, module implemented in GENEIOUS v 9.0.5) with default settings. We eliminated poorly aligned positions and divergent regions in the 12S and 16S gene alignments using GBLOCK 0.91b (Castresana, 2000).

We built a genetic dataset of 1,342 base pairs (bp) and 145 terminals representing 20 taxa. We partitioned this dataset according to genome and transcript type (12S 377 bp and 16S 545 bp ribosomal RNA; COI 420 bp protein-coding mRNA), subdividing protein-coding genes into codon position (1st; 2nd and 3rd codon position). We used PartitionFinder 2 with the Bayesian information criterion (BIC) to select the best-fit partitioning schemes and the most appropriate nucleotide replacement models (Lanfear et al., 2016) (Table 1). For phylogenetic analysis, we used the Bayesian inference implemented in MrBayes v3.2.6 (Ronquist et al., 2012) using the substitution models generated by PartitionFinder. We ran two independent runs of four Markov chains for 20 million generations sampling every 5000 generations and discarding 25% as burn-in. We evaluated the stability of the analysis in Tracer v1.6, ensuring that all ESS values were above 200 (Rambaut et al., 2014). We considered all posterior probabilities above 0.95 strongly supported. Additionally, we calculated sequence divergence of COI and 16S (uncorrected p-distance) among species/individuals of *Amazophrynella* using MEGA v7.0.26 (State College, PA) (Kumar, Stecher & Tamura, 2016). We used a threshold of 6% and 3% between COI and 16S barcodes, respectively, proposed for distinguishing Neotropical anuran species (Lyra, Haddad & Azeredo-Espin, 2017; Vences et al., 2005).

**Table 1:** Best-fitting partitioning scheme model of nucleotide substitution for 12S, 16S and COI mtDNA markers.

**Dataset**	**Partition**	**Base pairs**	**Model**
12S and 16S	12S, 16S	922	GTR+I+G+X
COI	COI 1st position	140	HKY+X
COI	COI 2nd position	140	TRNEF+G
COI	COI 3rd position	140	HKY+X

### Nomenclatural acts

The electronic edition of this article conforms to the requirements of the amended International Code of Zoological Nomenclature, and hence the new names contained herein are available under that Code of this article. This published work and the nomenclatural acts it contains have been registered in ZooBank, the online registration system for the ICZN. The LSID (Life Science Identifier) for this publication is: LSIDurn:lsid:zoobank.org:pub:11BFE482-B5D7-4371-A16A- CBBCBAAC3C3C. The electronic edition of this work was published in a journal with an ISSN, has been archived, and is available from the following digital repository: http://www.peerj.com/.

### Species delimitation

We followed the model proposed by Padial et al. (2010) to delimit the candidate species (CS). The CS can be classified as an Unconfirmed Candidate Species (UCS), Confirmed Candidate Species (CCS) and Deeply Conspecific Lineage (DCL). UCS is considered any CS that presents genetic differences above the limit (>3%) proposed for the 16S rRNA gene (Fouquet et al., 2007; Vences et al., 2005), but with no complementary characters verified, such as morphology, bioacoustics, ecology or distribution. A CCS is considered a CS that presents genetic differentiation in relation to other species and also has support of parallel evidence. Lastly, a DCL is considered a CS that presents genetic divergence above the proposed limit (>3%), but cannot be differentiated by parallel evidence (morphology and acoustics).

After observations of morphological characteristics of the population from Óbidos municipality, Pará state, we noticed a set of characters that distinguish these specimens from other species of the genus *Amazophrynella*. Using a complementary perspective (three mitochondrial genes), we were able to define this population as a CCS, based on genetic distance, phylogenetic position and morphology (see below).

## Results

**Figure 1:**
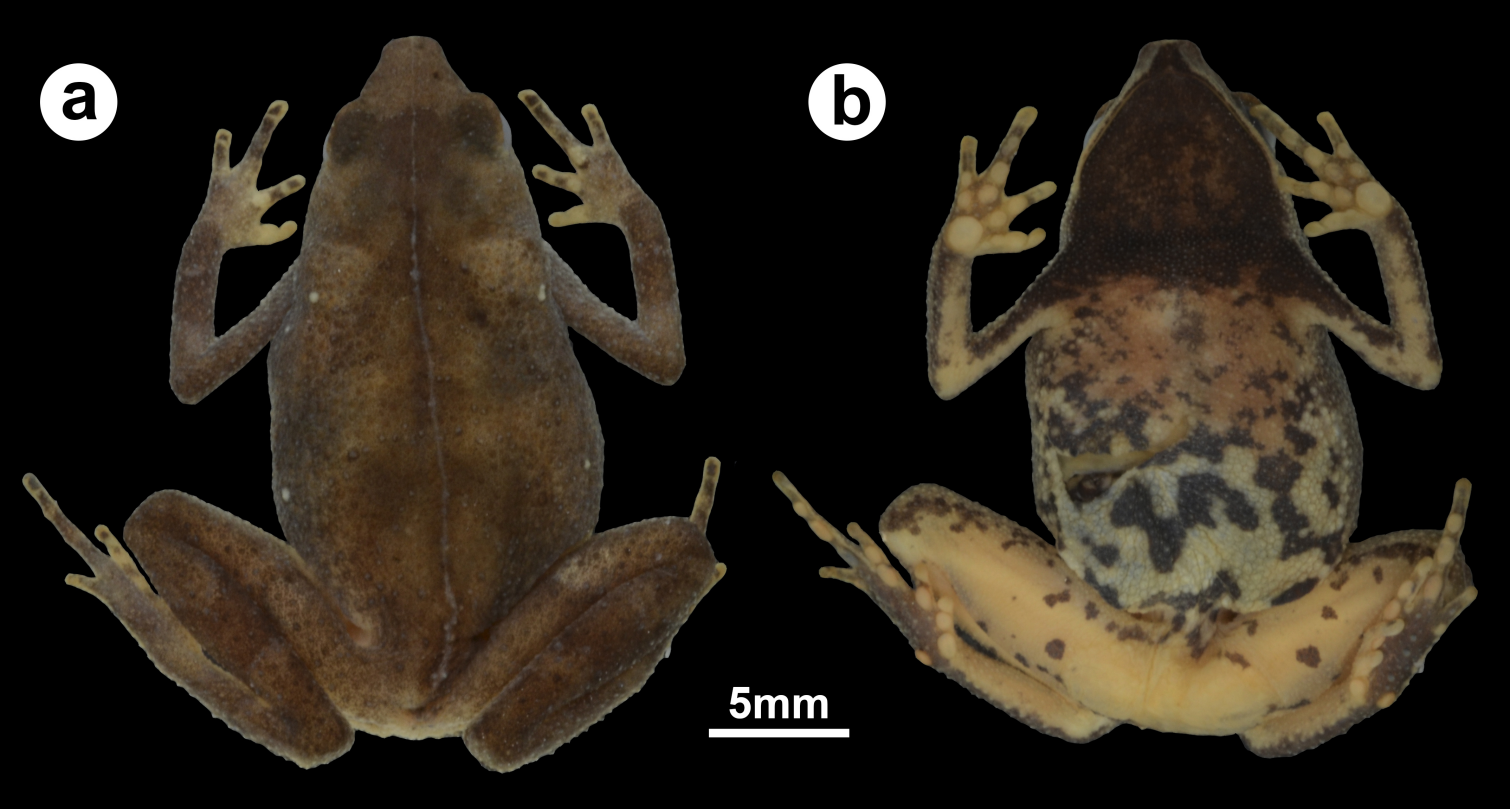
Holotype of *Amazophrynella gardai* sp. nov. (ZUFMS-AMP12821, adult female, SVL 24.4 mm) from Óbidos municipality, Pará state, Brazil. (A) Dorsal and (B) ventral views.

**Figure 2:**
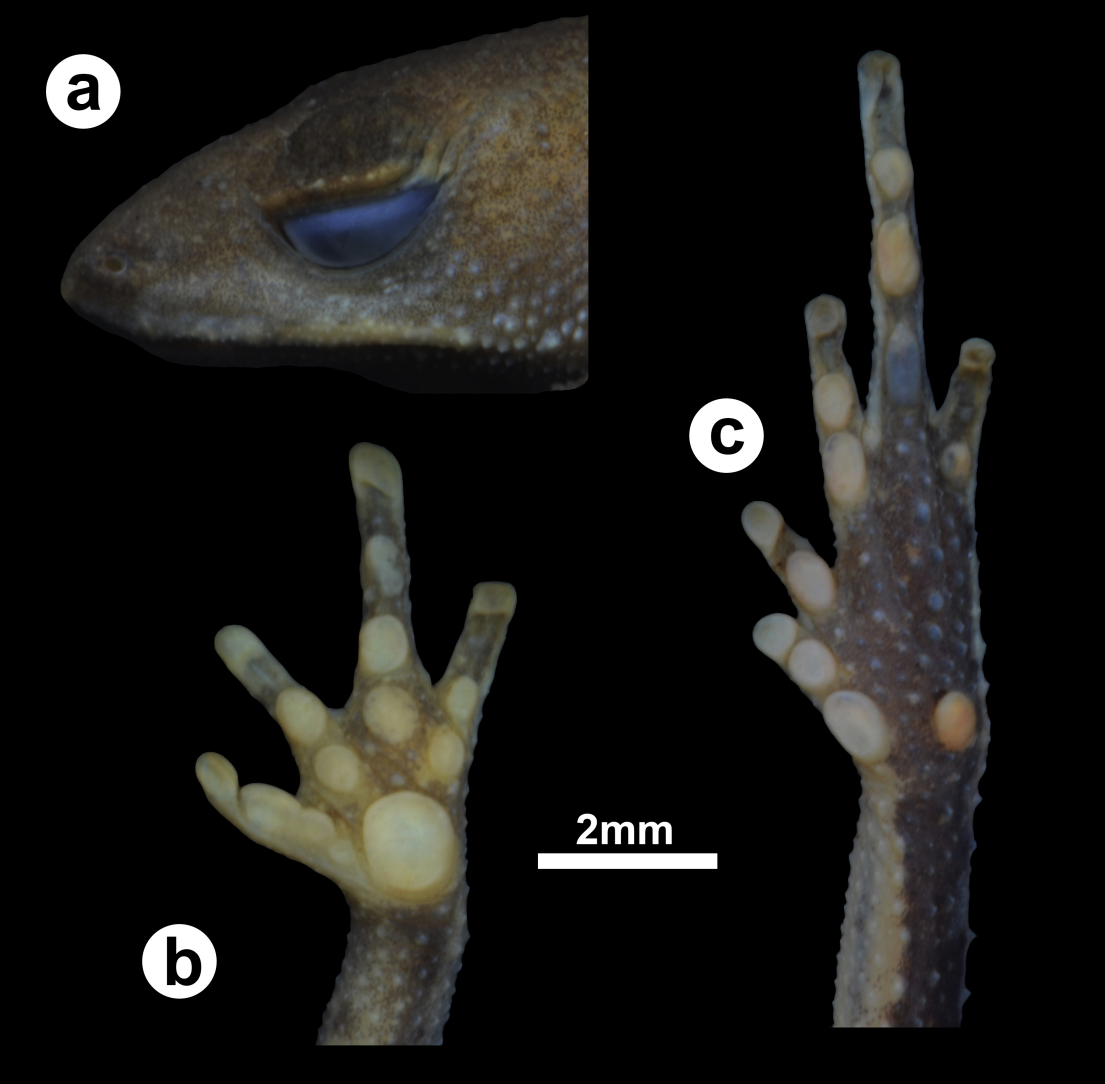
Holotype of *Amazophrynella gardai* sp. nov. (ZUFMS-AMP12821, adult female, SVL 12.15 mm) from Óbidos municipality, Pará state, Brazil. (A) lateral view of the head, ventral views of the (B) hand and the (C) foot.

**Figure 3:**
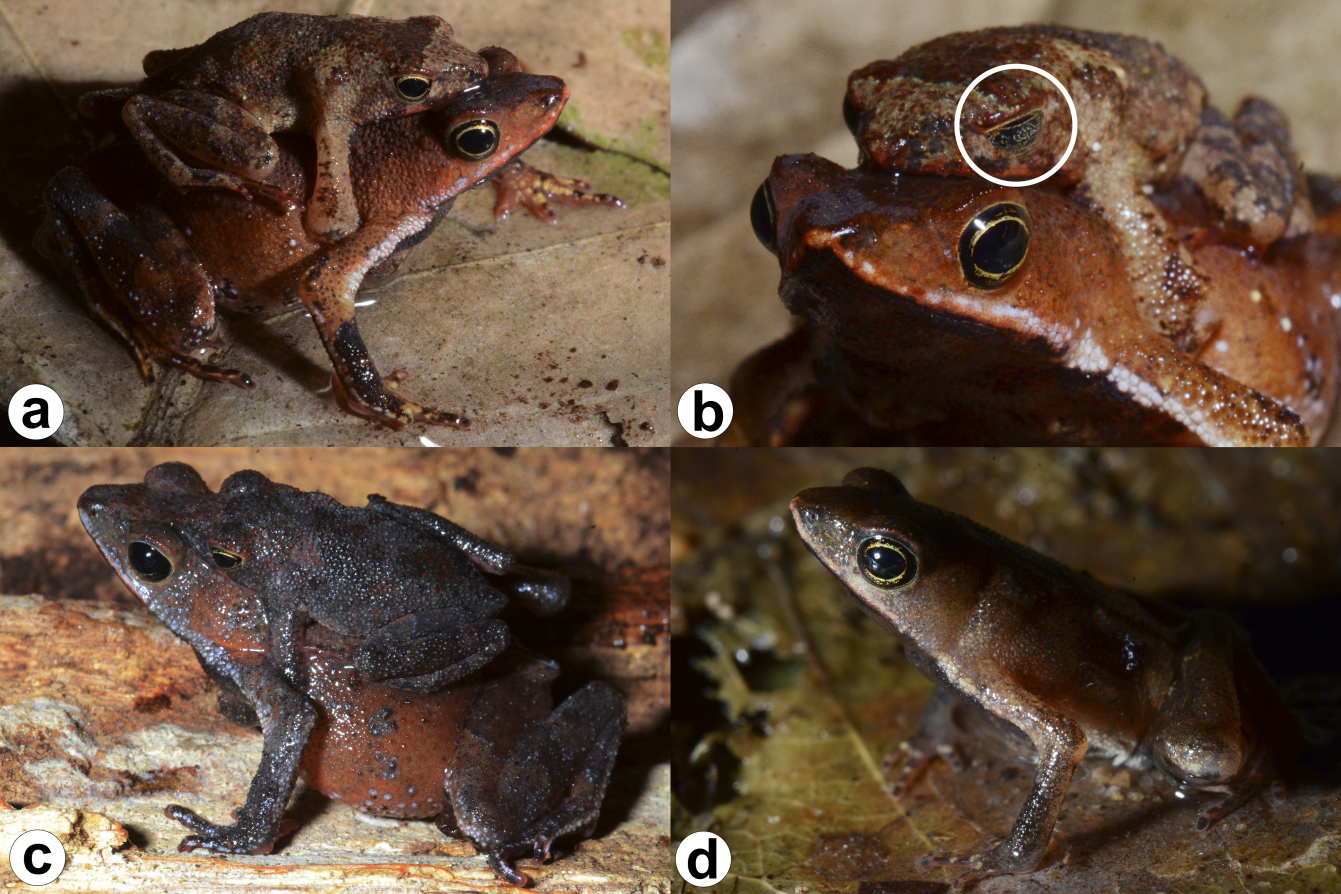
Live specimens of *Amazophrynella gardai* sp. nov. (A–B) Amplected couple (ZUFMS-AMP12827, adult male, SVL 16.0 mm; ZUFMS-AMP12826, adult female, 22.9 mm). (C) Amplected couple (ZUFMS-AMP12829, adult male, SVL 16.6 mm; ZUFMS-AMP12828, adult female, SVL 24.0 mm). (D) Adult male (ZUFMS-AMP12822, SVL 16.5 mm). White circle in the (B) indicates the reticulated lower eyelid.

**Figure 4:**
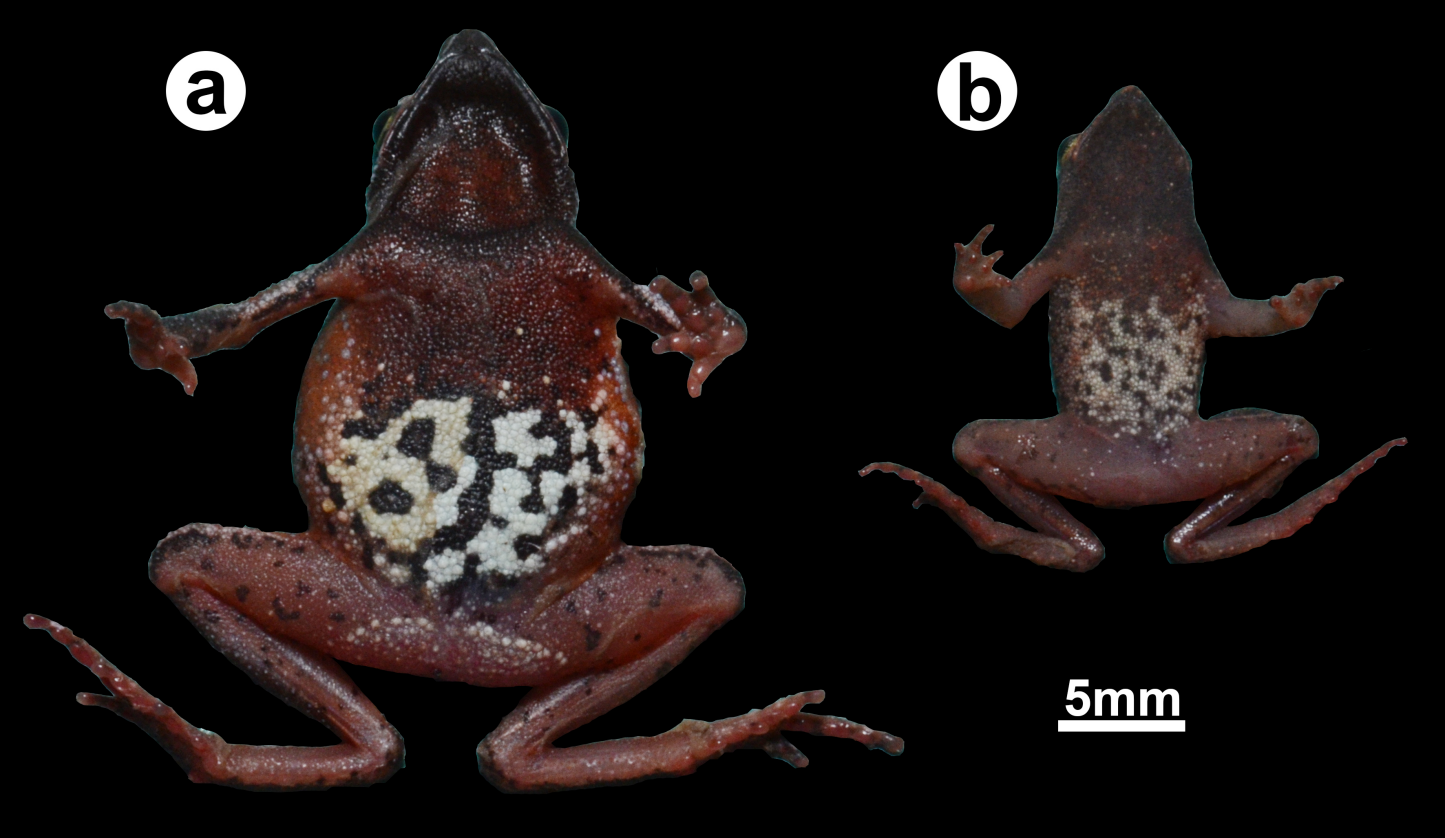
Ventral coloration in life. (A) a female (ZUFMS-AMP12829) and (B) a male (ZUFMS-AMP12828) of *Amazophrynella gardai* sp. nov.

**Figure 5:**
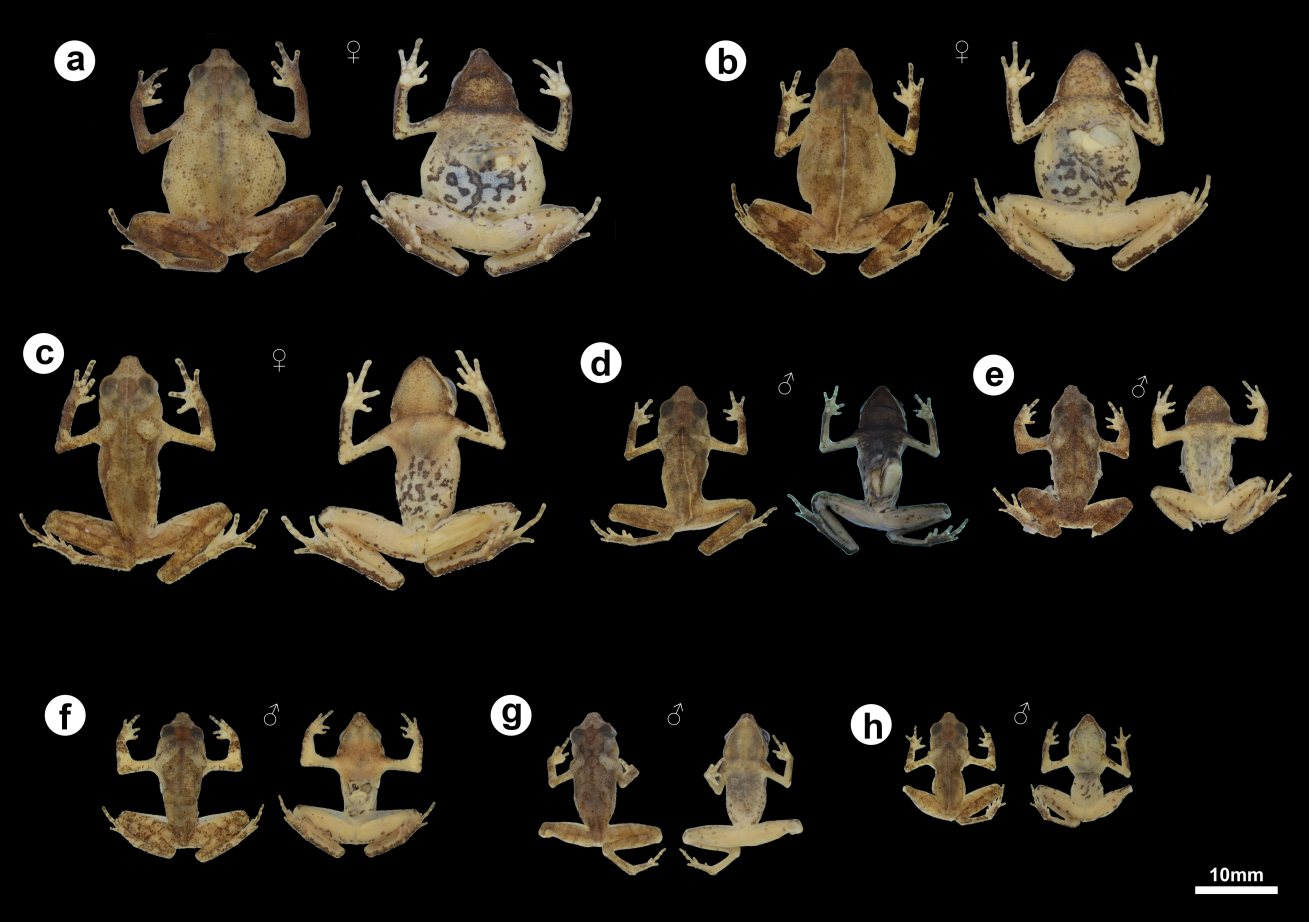
Morphological and color variation of preserved specimens of *Amazophrynella gardai* sp. nov., from Óbidos municipality, Pará state, Brazil. (A) ZUFMS-AMP12828 (SVL 24.0 mm), (B) ZUFMS-AMP12826 (SVL 22.9 mm), (C) ZUFMS-AMP12825 (SVL 24.2 mm), (D) ZUFMS-AMP12822 (SVL 16.5 mm), (E) ZUFMS-AMP12829 (SVL 16.6 mm), (F) ZUFMS-AMP12827 (SVL 16.0 mm), (G) ZUFMS-AMP12824 (SVL 17.8 mm), (H) ZUFMS-AMP12823 (SVL 12.6 mm, juvenile).

*Amazophrynella gardai* sp. nov. (Figs. 1–5, Table 1)

ZooBank LSID: urn:lsid:zoobank.org:pub:11BFE482-B5D7-4371-A16A-CBBCBAAC3C3C

**Holotype.** ZUFMS-AMP12821 (field number MAP 5758), adult female, collected at Óbidos municipality, Pará state, Brazil (datum = WGS84, 1°51′26″S; 55°32′55″W, ∼75 m a.s.l.), on 30 January 2015, by D.J. Santana.

**Paratypes.** ZUFMS-AMP12822, ZUFMS-AMP12824 (field numbers, MAP 5761, MAP 5759, respectively), adult males, ZUFMS-AMP12825 (field number, MAP 5765), adult female, collected at Óbidos municipality, Pará state, Brazil (datum = WGS84, 1°51′26″S; 55°32′55″W, ∼75 m a.s.l.), on 29 January 2015, by D.J. Santana. ZUFMS-AMP12823 (field number MAP 5764), juvenile, ZUFMS-AMP12827, ZUFMS-AMP12829 (field numbers MAP 5766, MAP 5760, respectively), adult males, ZUFMS-AMP12826, ZUFMS-AMP12828 (field numbers, MAP 5763, MAP 5762, respectively), adult females, collected at Óbidos municipality, Pará state, Brazil (datum = WGS84, 1°47′56″S; 55°34′27″W, ∼80 m a.s.l.), on 02 February 2015, by D.J. Santana.

**Diagnosis.** The new species can be distinguished using the following combination of traits: (1) large size for the genus (SVL of males 16.0–17.8 mm and females 22.9–24.4 mm); (2) snout elongated, acuminated in lateral view and truncated in dorsal view; (3) dorsal skin spiculated (small sized warts with pointed tips); (4) ventral region with dark brown blotches, with a white background; (5) palmar and subarticular tubercles rounded; (6) big palmar tubercle, occupying 2/4 of the palmar surface.

**Comparison with other species.** We compared the traits based on the analysis of individuals housed in collections (*A. manaos*, *A. matses*, *A. teko*, *A. vote*, and *A. xinguensis*), and on the literature available for the remaining species of the genus. Characteristics of compared species are presented in parentheses.

*Amazophrynella gardai* sp. nov. diagnosed from all species of the genus by its larger size: SVL 16.0–17.8 mm in males, *n* = 4, and 22.9–24.4 mm in females, *n* = 4 (*A. amazonicola*: 11.6–15.6 mm in males, 16.2–20.9 mm in females; *A. bilinguis*: 13.0–14.5 mm in males, 19.6–20.4 mm in females, Kaefer et al., 2019; *A. bokermanni*: 15.9–16.5 mm in males; *A. javierbustamantei*: 12.8–16.4 mm in males, 16.4–22.5 mm in females; *A. manaos*: 13.5–15.0 mm in males*; A. matses*: 11.5–13.5 mm in males; 15.6–19.0 mm in females; *A. minuta*: 12.2–14.7 mm in males, 14.5–19.4 mm in females; *A. moiseisii*: 12.2–16.9 mm in males, 16.4–20.9 mm in females; *A. siona*: 11.5–14.8 mm in males, 16.1–20.8 mm in females; *A. teko*: 12.9–15.8 mm in males, 17.9–21.6 mm in females, Rojas et al., 2018a), except for *A. vote*: (15.2–19.3 mm in males, 21.4–25.7 in females, Ávila et al., 2012), *A. xinguensis* (17.7–20 mm in males; 22.4–26.3 mm in females; Rojas et al., 2018a), and females of *A. manaos* (16.0–24.7 mm in females).

By the size of the palmar tubercle, occupying 2/4 of the palmar surface, *A. gardai* sp. nov. differs from all congeners, except for *A. bilinguis, A. teko* and *A. xinguensis* (palmar tubercle occupies 1/4 of the palmar surface in *A. amazonicola*, *A. bokermanni*, *A. javierbustamantei*, *A. manaos*, *A. matses*, *A. minuta*, *A. moisesii*, *A. siona*, and *A. vote*), and by the presence of rounded palmar surface, differs from *A. manaos, A. minuta*, A*. javierbustamantei*, *A. moisesii* and *A. teko* (elliptical), and *A. xinguensis* (ovoid). By the presence of finger I shorter than finger II, *A. gardai* sp. nov. differs from *A. bilinguis*, *A. bokermanni*, and *A. xinguensis* (FI>FII or FI=FII, Rojas et al., 2018a). *Amazophrynella gardai* sp. nov. also differs from all species of the genus by the dorsal skin spiculated (tuberculate or granular in its congeners). The ventral color surface white in life, with small dark brown blotches in *A. gardai* sp. nov. distinguish it from *A. amazonicola*, *A. javierbustamantei*, *A. minuta*, *A. matses* (venter yellow-orange with large, medium size or small blotches), *A. moisesii* (venter pale yellow, with small irregular dots), *A siona* (venter reddish brown, yellow blotches), *A. teko* (venter cream or yellow, with small blotches), *A. vote* (venter reddish brown, small dots), *A. xinguensis* (venter light gray, with tiny points).

According to the genetic analyses (see Phylogenetic relationships topic), the new species is more closely related to *A. manaos*, *A. teko*, and *A.* sp.1. *Amazophrynella gardai* sp. nov. differs from these three species by (1) having SVL 16.0–17.8 mm in males and 22.9–24.4 mm in females (*A. manaos*: 13.5–15.0 mm in males; 16.0–24.7 mm in females; *A. teko*: 12.9–15.8 mm in males, 17.9–21.6 mm in females, Rojas et al., 2018a), (2) presence of dorsal skin spiculated (*A. manaos*: granular; *A. teko* and *A.* sp1: highly granular, Rojas et al., 2018a), (3) presence of rounded palmar surface (elliptical; Rojas et al., 2018a). The new species also can be distinguished from *A. teko* and *A.* sp.1 by the presence of truncated snout and venter coloration white (snout acute and venter coloration creamy, Rojas et al., 2018a), and from *A. manaos*, by the size of the palmar tubercle, occupying 2/4 of the palmar surface (occupies 1/4 of the palmar surface).

**Description of the holotype.** Body small, elongated. Head triangular in dorsal and ventral views, wider than long (HL 28% and HW 26% of SVL). Snout elongated, acuminated in lateral view and truncated in dorsal view, SL 31% of HL. Nostrils protuberant, closer to snout than eyes. Internarial distance smaller than eye diameter, IND 26% of HW. Upper eyelid covered by small granules. Eye prominent, 33% of HL, presence of a reticulated lower eyelid. Tympanum not visible through the skin. Skin on tympanum covered with tubercles. Dorsal skin spiculated. Abundance of tubercles on the arms and legs. UAL 20% of SVL. UAL 85% of HAL. Fingers slender. Tips unexpanded. Fingers basally webbed. Relative length of Fingers: I <  II <  IV <  III. Supernumerary tubercles rounded: four located in the palm, and the first one is fused with the subarticular of Finger I. Subarticular tubercles are under the joints between falanges: one in Finger I, II and IV, and two in Finger III. Palmar tubercle rounded, around $ \frac{2}{4} $ of the palmar surface. Gular region and texture of the ventral skin tuberculated. Cloacal opening slightly above midlevel of thighs. Hind limbs slender. Thigh to tarsus covered by spiny protuberances. THL 53% of SVL. TAL 24% of SVL. FL 75% of THL. Relative length of toes: I <  II <  III <  V <  IV. Outer metatarsal tubercle small and rounded. Subarticular tubercles rounded in toes I and V, elliptical in II, III and IV: one in toes II and V, two in toe III, and three in toe IV; foot with slender, basally webbed toes. Color in preservative of the ventral surface pattern (dark brown blotches) is the same as described in life (see topic species coloration in life). The dorsal surface coloration became pale brown.

**Measurements of the holotype (mm).** SVL: 24.4, HL: 6.8, HW: 6.3, ED: 2.3, IND: 1.6, SL: 2.1, FI: 1.5, FII: 1.9, HAL: 5.6, UAL: 4.8, THL: 12.9, TL: 11.6, TAL: 5.8, FL: 9.6.

**Species coloration in life (Figs. 3 and 4).** Females exhibit a reddish dorsal color, with a distinct white and thin vertebral line (from the tip of the snout to cloaca). On lateral view, a white line extends from the tip of the snout, through the lip and the prolateral surface of the upper arm, to the elbow joint. A few small white dots on the flanks and dorsal region of the legs. Venter with large black blotches partially interconnected over light background. Males have dark brown or dark grey dorsal color; Ventral surface is predominantly cream-colored with small- and medium-size black blotches.

Variation: (Fig. 5). Only one male does not exhibit the thin white vertebral line (ZUFMS-AMP12822). Gular region varies from dark to light brown in both males and females. Morphometric variation for the type series is provided in Table 2.

**Table 2:** Morphometric measurements (mm) for the series type specimens of *Amazophrynella gardai* sp. nov. from Óbidos municipality, Pará state, Brazil. Abbreviations are defined in the Methods. Values (in mm) are presented as mean ± SD (range).

Measure	Males (*n* = 4)	Females (*n* = 4)
SLV	16.72 ± 0.75 (16.01–17.77)	23.86 ± 0.66 (22.89–24.37)
HL	4.37 ± 0.41 (4.04–4.92)	6.67 ± 0.20 (6.37–6.81)
HW	4.45 ± 0.47 (4.16–5.15)	6.22 ± 0.05 (6.17–6.28)
ED	1.84 ± 0.14 (1.66–2.01)	2.30 ± 0.12 (2.21–2.47)
IND	1.22 ± 0.08 (1.11–1.31)	1.67 ± 0.07 (1.62–1.76)
FI	0.64 ± 0.10 (0.52–0.72)	1.39 ± 0.21 (1.16–1.64)
FII	0.92 ± 0.07 (0.83–1.01)	1.75 ± 0.25 (1.54–2.04)
SL	1.70 ± 0.17 (1.52–1.90)	2.34 ± 0.15 (2.13–2.50)
HAL	3.46 ± 0.13 (3.31–3.60)	5.78 ± 0.31 (5.43–6.05)
UAL	3.81 ± 0.48 (3.24–4.38)	4.72 ± 0.60 (4.25–5.54)
THL	8.06 ± 0.91 (6.87–9.03)	12.58 ± 0.72 (11.54–13.16)
TL	7.59 ± 0.61 (6.84–8.18)	11.64 ± 0.69 (10.85–12.54)
TAL	4.46 ± 0.29 (4.14–4.84)	6.17 ± 0.61 (5.58–6.95)
FL	5.85 ± 0.50 (5.26–6.43)	9.54 ± 0.44 (8.94–9.99)

**Figure 6:**
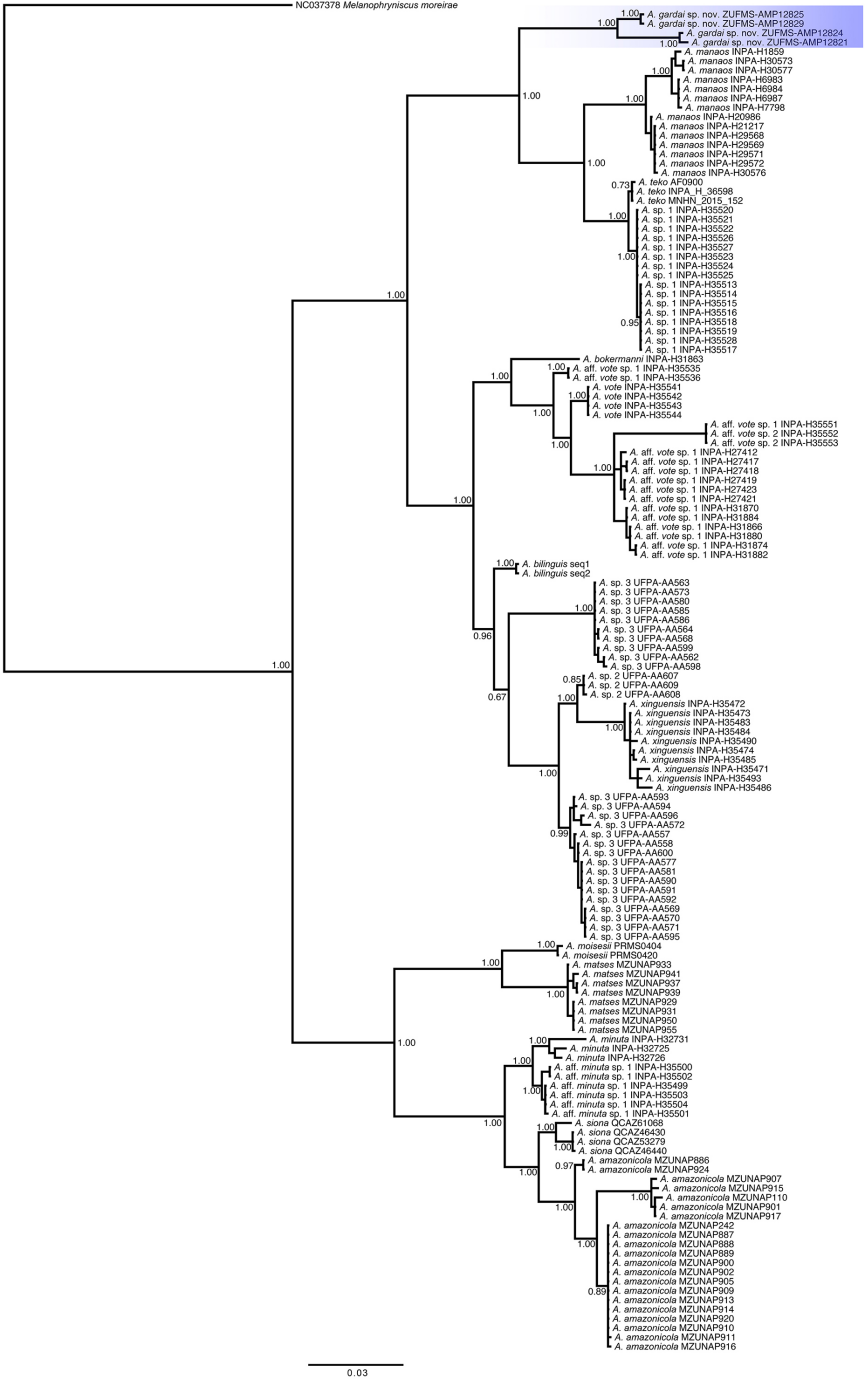
Phylogenetic relationships of species of the genus *Amazophrynella* based on analysis of the COI, 16S and 12S rDNA mitochondrial genes. Bayesian posterior probabilities are shown near nodes.

**Phylogenetic relationships (Fig. 6).** We recovered three main distinct clades in our phylogenetic analysis. The first clade is composed by *A. gardai* sp. nov., *A. teko*, *A. manaos*, and *Amazophrynella* sp.1, with *A. gardai* as sister taxon of the other three species. We found *A. teko* as paraphyletic in relation to *Amazophrynella* sp1., forming a sister taxa of *A. manaos*. The second main clade is composed by two subclades. The first one composed by *A. bokermanni*, which is sister taxon of *A. vote*, *A*. aff. *vote* sp.1, *A. vote*, and *A*. aff. *vote* sp.2. We recovered *A.* aff *vote* sp.1 as paraphyletic. The second subclade is formed by *A. bilinguis* as sister taxa of *A*. sp.2, *A. xinguensis* and *A*. sp.3, with the last being paraphyletic. The third main clade is formed by *A. moisesi* and *A. matses* as sister taxa of *A. minuta*, *A*. aff. *minuta* sp.1, *A. siona* and *A. amazonicola*. All species in this last clade are high supported.

The uncorrected p-distance of 16S and COI sequences between *A. gardai* sp. nov. and its sister clade was 5.6–8.1% and 8.8%, respectively. The intraspecific distance for *A. gardai* sp. nov. for COI was 2.3% and ranged from 0.0 to 4.9% in other species analyzed (Table S2 , Table S3).

**Figure 7:**
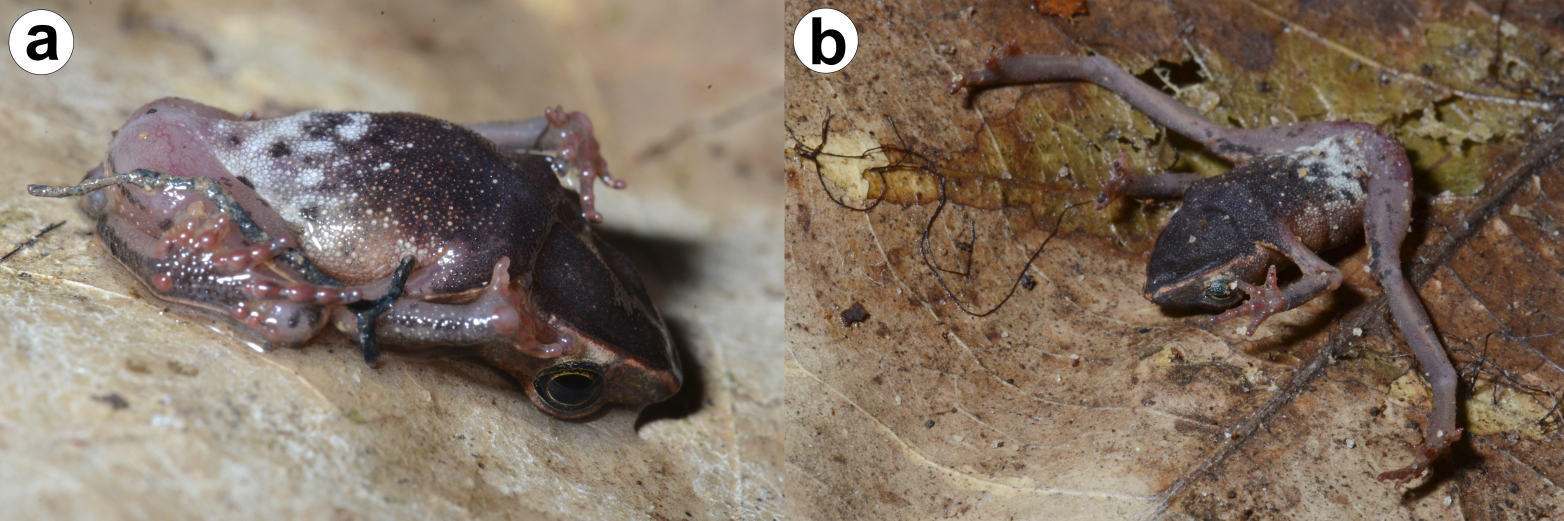
Defensive behavior on *Amazophrynella gardai* sp. nov. (A) Thanatosis and (B) Stiff-legged (ZUFMS-AMP12822, adult male, SVL 8.3 mm).

**Natural history (Fig. 7).** One male individual (ZUFMS-AMP12822) showed stiff-legged behavior and thanatosis (death-feigning) during manipulation for photographs. The stiff-legged behavior is a defensive strategy to avoid detection by predators and thanatosis is used to avoid subjugation (Bertoluci et al., 2007; Toledo, Sazima & Haddad, 2011). Russel (2002) documented death-feigning behavior in *Amazophrynella* “*minuta*” from Pacaya-Samiria National Reserve, Loreto, Peru (probably *A. matses* or *A. amazonicola*, see Rojas et al., 2015). This is the first report of stiff-legged behavior in the genus *Amazophrynella*.

We found individuals of *Amazophrynella gardai* sp. nov. by visual search and pitfall traps inside the forest (from 500 m up to 2,000 m from the edge). During visual search (both diurnal and nocturnal periods), we found three males (ZUFMS-AMP12822-24), one female (ZUFMS-AMP12821), and an amplected couple (ZUFMS-AMP12828-29) on the leaf litter. The individuals were in “*Terra firme*”, nearby to rivulets inside the forest, concentrating their activity during the morning, between 8:00–11:00 h am. Using the pitfall traps, we collected one female (ZUFMS-AMP12825) and one amplected couple (ZUFMS-AMP12826-27). February comprises the rainy season in the region and the presence of amplected couples may indicate that *Amazophrynela gardai* sp. nov. was in its breeding season. However, we have not observed males in calling activity.

**Etymology** The specific name is a patronym honoring Prof. Adrian Antonio Garda (Universidade Federal do Rio Grande do Norte, UFRN) for his extensive contributions to the knowledge of Neotropical anurans, his friendship, and his mentoring of SM and DJS during their doctorate degrees.

**Figure 8:**
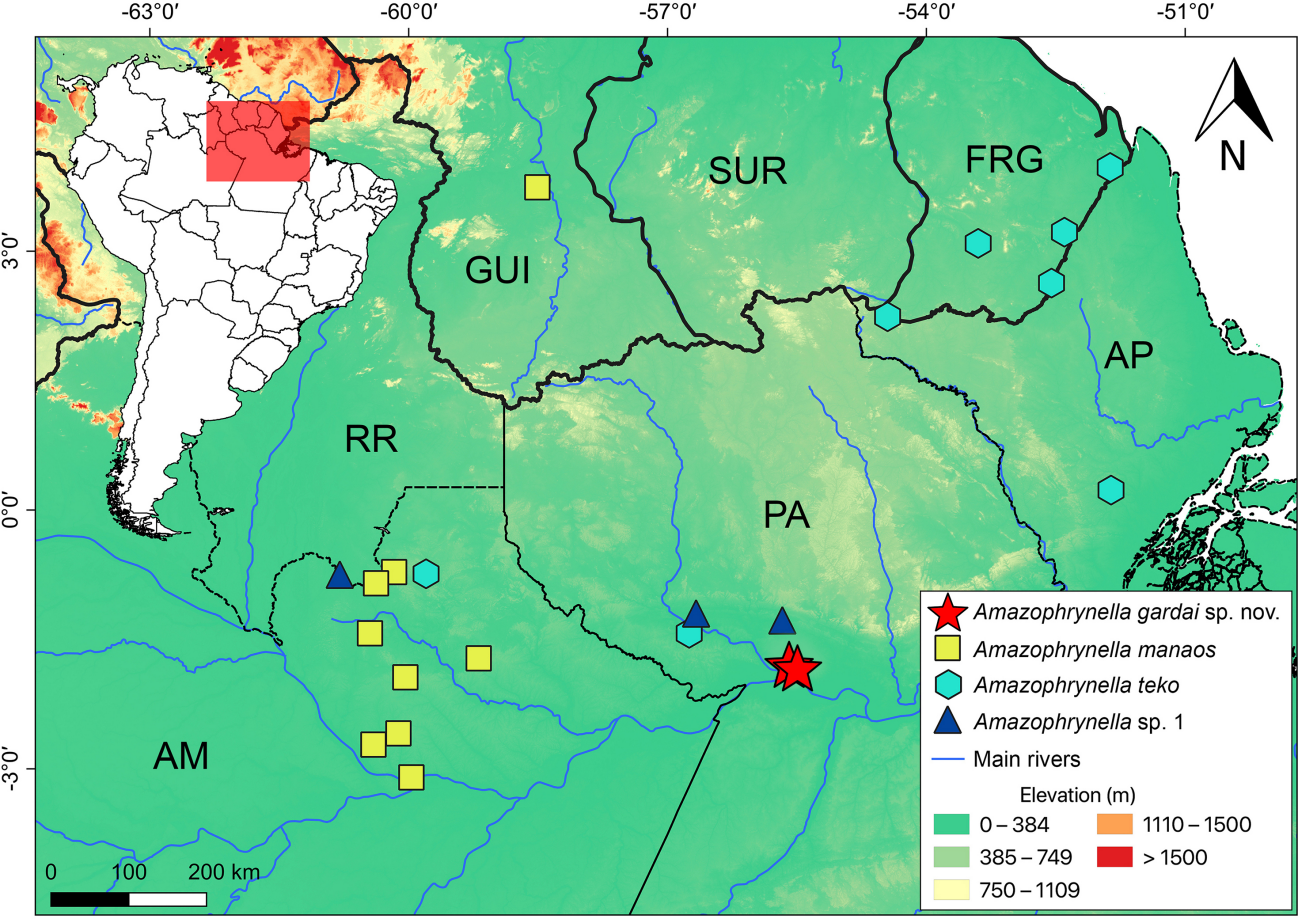
Geographic distribution of *Amazophrynella gardai* sp. nov., *A. manaos*, *A. teko*, and *A.* sp.1 in Amazonia. Countries: GUI, Guyana; SUR, Suriname; FRG, French Guyana, Brazilian states, AM, Amazonas; PA, Pará; AP, Amapá; RR, Roraima.

**Distribution (Fig. 8).** *Amazophynella gardai* sp. nov. is known only from its type locality, Óbidos municipality, Pará state, Brazil. The area where we found the new species is characterized as a Alluvial Forest type, with smaller trees where it is possible to observe a high concentration of palm trees.

## Discussion

In the last eight years, 10 new species of *Amazophrynella* have been described (see Kaefer et al., 2019; Frost, 2020), increasing the genus from two to 12 species (600%). This rapid rate of species description in addition to other recent studies have shown that the diversity of *Amazophrynella* is greatly underestimated (Rojas et al., 2018a; Kaefer et al., 2019). An aspect that underscores this situation is that the new species described here as well as the recently described, *A. bilinguis* (Kaefer et al., 2019), represent lineages in addition to the 11 recognized in Rojas et al. (2018a).

In our phylogenetic analyses we recovered the same three main clades observed by Rojas et al. (2018a). However, we found three previously recognized lineages (*A. teko*, *A.* aff. *vote* sp.1, and *A.* sp.3, Rojas et al., 2018a) as paraphyletic in our study. In addition to the *A. teko* paraphyletic in relation to *Amazophrynella* sp.1., we found 1.2% of 16S p-distances between these two lineages (1% in Kaefer et al., 2019, and 3% in Rojas et al., 2018a; Rojas et al., 2018b), which might indicate that they represent the same species. The difference between the topologies from Rojas et al. (2018a) and in the present work can be due to the different methods used in each study (concatenated *vs* concatenated with partition finder). We also recovered *A. bilinguis* as sister taxa of *A.* sp.2, *A.* sp.3, and *A. xinguensis*, different from the topology found by Kaefer et al. (2019), where *A. bilinguis* was recovered within a clade formed by *A.* sp.3, *A.* sp.2, and *A. xinguensis*. This result may be related to the use of a single gene (16S rRNA) in Kaefer et al. (2019). Given these differences found in the tree topologies, future analyzes including data from all species and using both mitochondrial and nuclear markers will better reveal the relationships among *Amazophrynella* species.

*Amazophrynella gardai* sp. nov., as well as the other recently described species, cannot even be considered a cryptic species because morphological characters easily distinguish it from its congeners. These findings emphasize the lack of studies in the region and highlight the importance of inventories in these areas, especially in the Guiana Shield, where many areas are still poorly known regarding the anuran fauna (Ávila Pires, Hoogmoed & Rocha, 2010).

All twelve *Amazophrynella* species are distributed in at least three sites (the same pattern was observed for the 11 candidate species found in Rojas et al., 2018a). *Amazophrynella gardai* sp. nov. occurs only in two very nearby sites (ca. 7 km straight-line distance), which we consider to represent a single population. However, its distribution may cover a larger area in the Guiana Shield after taxonomic re-evaluations of the populations in the region and/or new samplings in the area are conducted in order to verify this assumption.

## Conclusions

Based on genetic and morphological evidences, we described a new anuran species named *Amazophrynella gardai* sp. nov. The new species is one of the biggest within the genus and present a reticulated lower eyelid, large palmar tubercle and 5.6–8.1% uncorrected *p*-distance from its sister clade for the 16S mitochondrial gene, and 8.8% for the COI. The new species described here represents a newly discovered lineage, different from the other lineages already identified as candidate species and occurs only in two very nearby sites in the Pará state.

##  Supplemental Information

10.7717/peerj.9887/supp-1 Supplemental Information 1 Concatenated aligment of 12S, 16S and COI mtDNA

10.7717/peerj.9887/supp-2 Supplemental Information 2 Palmar tubercle (A) *Amazophrynella teko*; (B) *A. manaos*; (C) *A. gardai* sp. nov. Elliptical in (A) and (B); rounded in (C). Occupying 2/4 of the palmar surface in (A) and (C), and 1/4 in (B).

10.7717/peerj.9887/supp-3 Supplemental Information 3 Snout shape (A) *Amazophrynella teko*; acute in (B) *A. gardai* sp. nov.

10.7717/peerj.9887/supp-4 Supplemental Information 4 GenBank details: species and accession number

10.7717/peerj.9887/supp-5 Supplemental Information 5 Uncorrected p-distances for a 420-bp aligned sequence of the COI gene of the new species and 17 other *Amazophrynella* species (or candidate species) taken from GenBank (see Table S1) Data in bold are mean intraspecific divergences. N/C. Not Calculated

10.7717/peerj.9887/supp-6 Supplemental Information 6 Uncorrected p-distances for a 543-bp aligned sequence of the 16S gene of the new species and 17 other *Amazophrynella* species (or candidate species) taken from GenBank (see Table S1) Data in bold are mean intraspecific divergences. N/C. Not Calculated

10.7717/peerj.9887/supp-7 Supplemental Information 7 Raw Data morphometry of the type series

## Appendix

Specimens Examined

*Amazophrynella gardai* sp. nov.: BRAZIL: Pará: Óbidos: ZUFMS-AMP12821-29.

*Amazophrynella manaos*: BRAZIL: Amazonas: Presidente Figueiredo: INPA-H 29569, 29570, 29572 (paratypes).

*Amazophrynella matses*. PERU: Department Loreto: Requena, Nuevo Salvador: MZUNAP 936 (paratopotype); Jerano Herrera: MZUNAP 931 (paratype).

*Amazophrynella teko*. FRENCH GUIANA: Saunt Maripa: INPA-H 36597, 36599, 36600, 36601.

*Amazophrynella vote*. BRAZIL: Amazonas: INPA-H 27418, 27421, 27426 (paratypes).

*Amazophrynella xinguensis*. BRAZIL: Pará: Anapú, Sustainable Development Project (PDS) Virola Jatobá: INPA-H 35471 (holotype); Senador José Porfírio, Fazenda Paraiso: INPA-H 35493, 35490 (paratypes).

## Additional Information and Declarations

### DNA Deposition

The following information was supplied regarding the deposition of DNA sequences: Data is available at GenBank: MH270146, MH270147, MH270148, MH270149, MH270150, MH270151, MH270241, MH270242, MH270243, MH270244, MH270248, MH270249, MH270240, MH270239, MH270236, MH270237, MH270235, MH270238, MH270232, MH270233, MH270251, MH270252, MH270253, MH270139, MH270125, MH270118, MH270122, MH270120, MH270127, MH270119, MH270135, MH270121, MH270124, MH270138, MH270126, MH270132, MH270131, MH270129, MH270128, MH270136, MH270130, MH270140, MH270123, MH270117, MH270213, MH270205, MH270206, MH270293, MH270297, MH270298, MH270299, MH270300, MH270302, MH270303, MH270291, MH270090, MH270290, MH270294, MH270295, MH270288, MH270289, MH270180, MH270182, MH270183, MH270186, MH270187, MH270192, MH270185, MH270184, MH270170, MH270171, MH270160, MH270207, MH270208, MH270328, MH270327, MH270326, MH270329, MH270307, MH270308, MH270309, MH270310, MH270311, MH270312, MH270313, MH270314, MH270315, MH270316, MH270317, MH270318, MH270319, MH270320, MH270321, MH270322, MH270254, MH270255, MH270256, MH270258, MH270259, MH270221, MH270222, MH270223, MH270224, MH270268, MH270269, MH270270, MH270271, MH270225, MH270260, MH270226, MH270261, MH270227, MH270228, MH270262, MH270263, MH270264, MH270265, MH270266, MH270267, MH270272, MH270229, MH270230, MH270257, MH270325, MH270324, MH270323, MH270209, MH270210, MH270211, MH270212, MH270275, MH270274, MH270279, MH270280, MH270282, MH270277, MH270278, MH270283, MH270276, MH270281, KT221611, NC_037378, MH269954, MH269955, MH269956, MH269957, MH269958, MH269959, MH270029, MH270030, MH270031, MH270032, MH270036, MH270034, MH270027, MH270026, MH270023, MH270024, MH270022, MH270025, MH270019, MH270020, MH270037, MH270038, MH270039, KF681670, MH269940, MH269933, MH269937, MH269935, MH269942, MH269934, KF681668, MH269936, MH269939, KF681671, MH269941, MH269947, MH269946, MH269944, MH269943, KF681669, MH269945, KF681672, MH269938, MH269932, MH270081, MH270002, MH270003, KF433964, MH270082, MH270083, MH270084, MH270085, MH270087, MH270088, KF433961, MH270090, KF433959, KF433954, KF433955, KF433956, KF433958, MH269984, MH269985, MH269987, MH269990, MH269991, MH269992, MH269989, MH269988, MH269978, MH269979, MH269968, MH270004, MH270005, MH270113, MH270112, MH270111, MH270114, MH270091, MH270092, MH270093, MH270094, MH270095, MH270096, MH270097, MH270098, MH270099, MH270100, MH270101, MH270102, MH270103, MH270104, MH270105, MH270106, MH270041, MH270042, MH270043, MH270055, MH270056, MH270044, MH270045, MH270046, MH270047, MH270065, MH270066, MH270067, MH270068, MH270048, MH270057, MH270049, MH270058, MH270050, MH270051, MH270059, MH270060, MH270061, MH270062, MH270063, MH270064, MH270069, MH270052, MH270053, MH270054, MH270109, MH270108, MH270107, MH270007, MH270008, MH270009, MH270010, MH270071, MH270070, MH270075, MH270076, MH270078, MH270073, MH270074, MH270079, MH270072, MH270077, KT221611, NC_037378, MH269744, MH269745, MH269746, MH269747, MH269748, MH269749, MH269839, MH269840, MH269841, MH269842, MH269846, MH269847, MH269837, MH269836, MH269833, MH269834, MH269832, MH269835, MH269829, MH269830

MH269849, MH269850, MH269851, MH269737, MH269723, MH269716, MH269720, MH269718, MH269725, MH269717, MH269733, MH269719, MH269722, MH269736, MH269724, MH269730, MH269729, MH269727, MH269726, MH269734, MH269728, MH269738, MH269721, MH269715, MH269883, MH269802, MH269803, MH269892, MH269896,, MH269897, MH269898, MH269899, MH269901, MH269902, MH269890, MH270305, MH269889, MH269893, MH269894, MH269887, MH269888, MH269777, MH269778, MH269780, MH269783, MH269784, MH269785, MH269782, MH269781, MH269767, MH269768, MH269757, MH269804, MH269805, MH269928, MH269927, MH269926, MH269929, MH269906, MH269907, MH269908, MH269909, MH269910, MH269911, MH269912, MH269913, MH269914, MH269915, MH269916, MH269917, MH269918, MH269919, MH269920, MH269921, MH269852, MH269853, MH269854, MH269856, MH269857, MH269819, MH269820, MH269821, MH269822, MH269866, MH269867, MH269868, MH269869, MH269823, MH269858, MH269824, MH269859, MH269825, MH269826, MH269860, MH269861, MH269862, MH269863, MH269864, MH269865, MH269870, MH269827, MH269828, MH269855, MH269924, MH269923, MH269922, MH269807, MH269808, MH269809, MH269810, MH269873, MH269872, MH269877, MH269878, MH269880, MH269875, MH269876, MH269881, MH269874, MH269879, KT221611, NC_037378.

### Data Availability

The following information was supplied regarding data availability: The individuals used for describe the new species (type series) are housed at ”Coleção Zoológica da Universidade Federal de Mato Grosso do Sul (acronym ZUFMS-AMP)”: ZUFMS-AMP12821 (holotype) ZUFMS-AMP12822 ZUFMS-AMP12823 ZUFMS-AMP12824 ZUFMS-AMP12825 ZUFMS-AMP12826 ZUFMS-AMP12827 ZUFMS-AMP12828 ZUFMS-AMP12829

### New Species Registration

The following information was supplied regarding the registration of a newly described species: Publication LSID: urn:lsid:zoobank.org:pub:11BFE482-B5D7-4371-A16A- CBBCBAAC3C3C. *Amazophrynella* LSID: urn:lsid:zoobank.org:pub:11BFE482-B5D7-4371-A16A-CBBCBAAC3C3C. *gardai* LSID: urn:lsid:zoobank.org:pub:11BFE482-B5D7-4371-A16A-CBBCBAAC3C3C.
