# Supplementary figures and images for: A new tiny toad species of Amazophrynella (Anura: Bufonidae) from east of the Guiana Shield in Amazonia, Brazil
Source: PeerJ. 2020 Sep 18;8:e9887. doi: 10.7717/peerj.9887 (PMC7505081; doi:10.7717/peerj.9887)

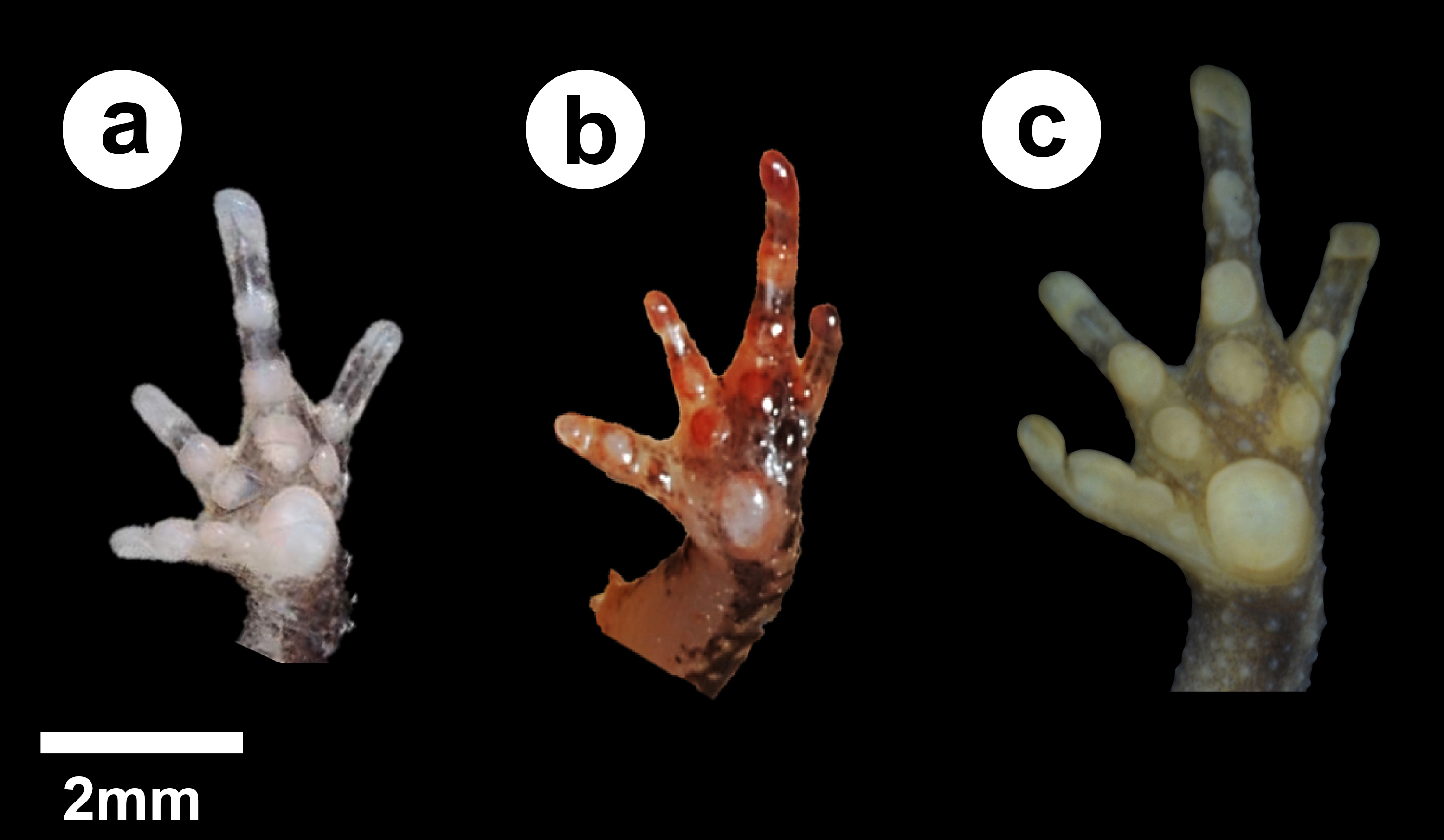

Supplement: Supplemental Information 2 — (A) Amazophrynella teko; (B) A. manaos; (C) A. gardai sp. nov. Elliptical in (A) and (B); rounded in (C). Occupying 2/4 of the palmar surface in (A) and (C), and 1/4 in (B). [file peerj-08-9887-s002.jpg]

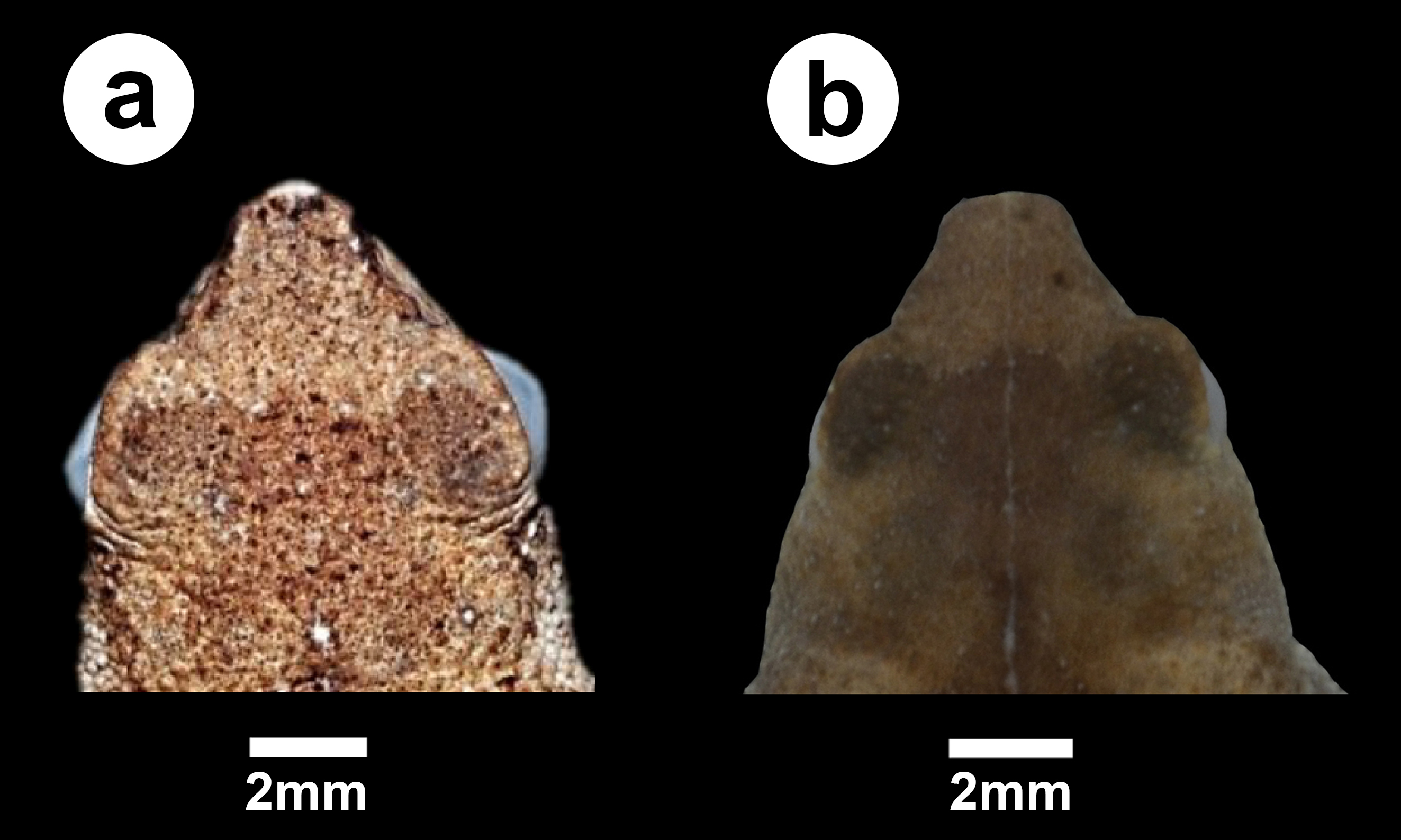

Supplement: Supplemental Information 3 — (A) Amazophrynella teko; acute in (B) A. gardai sp. nov. [file peerj-08-9887-s003.jpg]
