# Supplementary material for: A new tiny toad species of Amazophrynella (Anura: Bufonidae) from east of the Guiana Shield in Amazonia, Brazil
Source: PeerJ. 2020 Sep 18;8:e9887. doi: 10.7717/peerj.9887 (PMC7505081; doi:10.7717/peerj.9887)
Supplement: Supplemental Information 4 [file peerj-08-9887-s004.docx]

**Table S1.** GenBank details: Species and accession number.

| COI | 16S | 12S | Voucher number |
| --- | --- | --- | --- |
| MK682467 | - | - | *Amazophrynela* ***gardai*** sp. nov. ZUFMS-AMP12824 |
| MK682468 | MK684254 | MK684253 | *Amazophrynela* ***gardai*** sp. nov. ZUFMS-AMP12821 (holotype) |
| MK682469 | - | - | *Amazophrynela* ***gardai*** sp. nov. ZUFMS-AMP12825 |
| MK682470 | - | - | *Amazophrynela* ***gardai*** sp. nov. ZUFMS-AMP12829 |
| - | MK567965 | - | *Amazophrynela bilinguis* voucher INPAH H 39776 |
| - | MK567964 | - | *Amazophrynela bilinguis* voucher INPA H 39784 |
| MH270146 | MH269954 | MH269744 | *A.* aff. *minuta* sp. 1 RRR 2018 voucher INPA H 35499 |
| MH270147 | MH269955 | MH269745 | *A.* aff. *minuta* sp. 1 RRR 2018 voucher INPA H 35500 |
| MH270148 | MH269956 | MH269746 | *A.* aff. *minuta* sp. 1 RRR 2018 voucher INPA H 35501 |
| MH270149 | MH269957 | MH269747 | *A.* aff. *minuta* sp. 1 RRR 2018 voucher INPA H 35502 |
| MH270150 | MH269958 | MH269748 | *A.* aff. *minuta* sp. 1 RRR 2018 voucher INPA H 35503 |
| MH270151 | MH269959 | MH269749 | *A*. aff. *minuta* sp. 1 RRR 2018 voucher INPA H 35504 |
| MH270241 | MH270029 | MH269839 | *A*. aff. *vote* sp. 1 RRR 2018 voucher INPA H 27412 |
| MH270242 | MH270030 | MH269840 | *A.* aff. *vote* sp. 1 RRR 2018 voucher INPA H 27417 |
| MH270243 | MH270031 | MH269841 | *A.* aff. *vote* sp. 1 RRR 2018 voucher INPA H 27418 |
| MH270244 | MH270032 | MH269842 | *A.* aff. *vote* sp. 1 RRR 2018 voucher INPA H 27419 |
| MH270248 | MH270036 | MH269846 | *A.* aff. *vote* sp. 1 RRR 2018 voucher INPA H 27421 |
| MH270249 | MH270034 | MH269847 | *A*. aff. *vote* sp. 1 RRR 2018 voucher INPA H 27423 |
| MH270240 | MH270027 | MH269837 | *A.* aff. *vote* sp. 1 RRR 2018 voucher INPA H 31866 |
| MH270239 | MH270026 | MH269836 | *A*. aff. *vote* sp. 1 RRR 2018 voucher INPA H 31870 |
| MH270236 | MH270023 | MH269833 | *A.* aff. *vote* sp. 1 RRR 2018 voucher INPA H 31874 |
| MH270237 | MH270024 | MH269834 | *A.* aff. *vote* sp. 1 RRR 2018 voucher INPA H 31880 |
| MH270235 | MH270022 | MH269832 | *A.* aff. *vote* sp. 1 RRR 2018 voucher INPA H 31882 |
| MH270238 | MH270025 | MH269835 | *A*. aff. *vote* sp. 1 RRR 2018 voucher INPA H 31884 |
| MH270232 | MH270019 | MH269829 | *A*. aff. *vote* sp. 1 RRR 2018 voucher INPA H 35535 |
| MH270233 | MH270020 | MH269830 | *A.* aff. *vote* sp. 1 RRR 2018 voucher INPA H 35536 |
| MH270251 | MH270037 | MH269849 | *A.* aff. *vote* sp. 1 RRR 2018 voucher INPA H 35551 |
| MH270252 | MH270038 | MH269850 | *A*. aff. *vote* sp. 2 RRR 2018 voucher INPA H 35552 |
| MH270253 | MH270039 | MH269851 | *A*. aff. *vote* sp. 2 RRR 2018 voucher INPA H 35553 |
| MH270139 | KP681670 | MH269737 | *A*. *amazonicola* voucher MZUNAP:110 |
| MH270125 | MH269940 | MH269723 | *A*. *amazonicola* voucher MZUNAP:242 |
| MH270118 | MH269933 | MH269716 | *A*. *amazonicola* voucher MZUNAP:886 |
| MH270122 | MH269937 | MH269720 | *A*. *amazonicola* voucher MZUNAP:887 |
| MH270120 | MH269935 | MH269718 | *A*. *amazonicola* voucher MZUNAP:888 |
| MH270127 | MH269942 | MH269725 | *A*. *amazonicola* voucher MZUNAP:889 |
| MH270119 | MH269934 | MH269717 | *A*. *amazonicola* voucher MZUNAP:900 |
| MH270135 | KP681668 | MH269733 | *A*. *amazonicola* voucher MZUNAP:901 |
| MH270121 | MH269936 | MH269719 | *A*. *amazonicola* voucher MZUNAP:902 |
| MH270124 | MH269939 | MH269722 | *A*. *amazonicola* voucher MZUNAP:905 |
| MH270138 | KP681671 | MH269736 | *A*. *amazonicola* voucher MZUNAP:907 |
| MH270126 | MH269941 | MH269724 | *A*. *amazonicola* voucher MZUNAP:909 |
| MH270132 | MH269947 | MH269730 | *A*. *amazonicola* voucher MZUNAP:910 |
| MH270131 | MH269946 | MH269729 | *A*. *amazonicola* voucher MZUNAP:911 |
| MH270129 | MH269944 | MH269727 | *A*. *amazonicola* voucher MZUNAP:913 |
| MH270128 | MH269943 | MH269726 | *A*. *amazonicola* voucher MZUNAP:914 |
| MH270136 | KP681669 | MH269734 | *A*. *amazonicola* voucher MZUNAP:915 |
| MH270130 | MH269945 | MH269728 | *A*. *amazonicola* voucher MZUNAP:916 |
| MH270140 | KP681672 | MH269738 | *A*. *amazonicola* voucher MZUNAP:917 |
| MH270123 | MH269938 | MH269721 | *A*. *amazonicola* voucher MZUNAP:920 |
| MH270117 | MH269932 | MH269715 | *A*. *amazonicola* voucher MZUNAP:924 |
| MH270213 | MH270081 | MH269883 | *A*. *bokermanni* voucher INPA H 31863 |
| MH270293 | KF433964 | MH269892 | *A*. *manaos* voucher INPA H 1859 |
| MH270297 | MH270082 | MH269896 | *A*. *manaos* voucher INPA H 20986 |
| MH270298 | MH270083 | MH269897 | *A*. *manaos* voucher INPA H 21217 |
| MH270299 | MH270084 | MH269898 | *A*. *manaos* voucher INPA H 29568 |
| MH270300 | MH270085 | MH269899 | *A*. *manaos* voucher INPA H 29569 |
| MH270302 | MH270087 | MH269901 | *A*. *manaos* voucher INPA H 29571 |
| MH270303 | MH270088 | MH269902 | *A*. *manaos* voucher INPA H 29572 |
| MH270291 | KF433961 | MH269890 | *A*. *manaos* voucher INPA H 30573 |
| MH270090 | MH270090 | MH270305 | *A*. *manaos* voucher INPA H 30576 |
| MH270290 | KF433959 | MH269889 | *A*. *manaos* voucher INPA H 30577 |
| MH270294 | KF433954 | MH269893 | *A*. *manaos* voucher INPA H 6983 |
| MH270295 | KF433955 | MH269894 | *A*. *manaos* voucher INPA H 6984 |
| MH270288 | KF433956 | MH269887 | *A*. *manaos* voucher INPA H 6987 |
| MH270289 | KF433958 | MH269888 | *A*. *manaos* voucher INPA H 7798 |
| MH270180 | MH269984 | MH269777 | *A*. *matses* voucher MZUNAP:929 |
| MH270182 | MH269985 | MH269778 | *A*. *matses* voucher MZUNAP:931 |
| MH270183 | MH269987 | MH269780 | *A*. *matses* voucher MZUNAP:933 |
| MH270186 | MH269990 | MH269783 | *A*. *matses* voucher MZUNAP:937 |
| MH270187 | MH269991 | MH269784 | *A*. *matses* voucher MZUNAP:939 |
| MH270192 | MH269992 | MH269785 | *A*. *matses* voucher MZUNAP:941 |
| MH270185 | MH269989 | MH269782 | *A*. *matses* voucher MZUNAP:950 |
| MH270184 | MH269988 | MH269781 | *A*. *matses* voucher MZUNAP:955 |
| MH270170 | MH269978 | MH269767 | *A*. *minuta* voucher INPA H 32725 |
| MH270171 | MH269979 | MH269768 | *A*. *minuta* voucher INPA H 32726 |
| MH270160 | MH269968 | MH269757 | *A*. *minuta* voucher INPA H 32731 |
| MH270207 | MH270004 | MH269804 | *A*. *moisesii* voucher PRMS 0404 |
| MH270208 | MH270005 | MH269805 | *A.* *moisesii* voucher PRMS 0420 |
| MH270328 | MH270113 | MH269928 | *A*. *siona* voucher QCAZ:46430 |
| MH270327 | MH270112 | MH269927 | *A*. *siona* voucher QCAZ:46440 |
| MH270326 | MH270111 | MH269926 | *A*. *siona* voucher QCAZ:53279 |
| MH270329 | MH270114 | MH269929 | *A*. *siona* voucher QCAZ:61068 |
| MH270307 | MH270091 | MH269906 | *A*. sp. 1 RRR 2018 voucher INPA H 35513 |
| MH270308 | MH270092 | MH269907 | *A*. sp. 1 RRR 2018 voucher INPA H 35514 |
| MH270309 | MH270093 | MH269908 | *A*. sp. 1 RRR 2018 voucher INPA H 35515 |
| MH270310 | MH270094 | MH269909 | *A*. sp. 1 RRR 2018 voucher INPA H 35516 |
| MH270311 | MH270095 | MH269910 | *A*. sp. 1 RRR 2018 voucher INPA H 35517 |
| MH270312 | MH270096 | MH269911 | *A*. sp. 1 RRR 2018 voucher INPA H 35518 |
| MH270313 | MH270097 | MH269912 | *A*. sp. 1 RRR 2018 voucher INPA H 35519 |
| MH270314 | MH270098 | MH269913 | *A*. sp. 1 RRR 2018 voucher INPA H 35520 |
| MH270315 | MH270099 | MH269914 | *A*. sp. 1 RRR 2018 voucher INPA H 35521 |
| MH270316 | MH270100 | MH269915 | *A*. sp. 1 RRR 2018 voucher INPA H 35522 |
| MH270317 | MH270101 | MH269916 | *A*. sp. 1 RRR 2018 voucher INPA H 35523 |
| MH270318 | MH270102 | MH269917 | *A*. sp. 1 RRR 2018 voucher INPA H 35524 |
| MH270319 | MH270103 | MH269918 | *A*. sp. 1 RRR 2018 voucher INPA H 35525 |
| MH270320 | MH270104 | MH269919 | *A.* sp. 1 RRR 2018 voucher INPA H 35526 |
| MH270321 | MH270105 | MH269920 | *A*. sp. 1 RRR 2018 voucher INPA H 35527 |
| MH270322 | MH270106 | MH269921 | *A*. sp. 1 RRR 2018 voucher INPA H 35528 |
| MH270254 | MH270041 | MH269852 | *A*. sp. 2 RRR 2018 voucher UFPA AA 607 |
| MH270255 | MH270042 | MH269853 | *A*. sp. 2 RRR 2018 voucher UFPA AA 608 |
| MH270256 | MH270043 | MH269854 | *A*. sp. 2 RRR 2018 voucher UFPA AA 609 |
| MH270258 | MH270055 | MH269856 | *A*. sp. 3 RRR 2018 voucher UFPA AA 557 |
| MH270259 | MH270056 | MH269857 | *A*. sp. 3 RRR 2018 voucher UFPA AA 558 |
| MH270221 | MH270044 | MH269819 | *A*. sp. 3 RRR 2018 voucher UFPA AA 562 |
| MH270222 | MH270045 | MH269820 | *A*. sp. 3 RRR 2018 voucher UFPA AA 563 |
| MH270223 | MH270046 | MH269821 | *A*. sp. 3 RRR 2018 voucher UFPA AA 564 |
| MH270224 | MH270047 | MH269822 | *A*. sp. 3 RRR 2018 voucher UFPA AA 568 |
| MH270268 | MH270065 | MH269866 | *A*. sp. 3 RRR 2018 voucher UFPA AA 569 |
| MH270269 | MH270066 | MH269867 | *A*. sp. 3 RRR 2018 voucher UFPA AA 570 |
| MH270270 | MH270067 | MH269868 | *A*. sp. 3 RRR 2018 voucher UFPA AA 571 |
| MH270271 | MH270068 | MH269869 | *A*. sp. 3 RRR 2018 voucher UFPA AA 572 |
| MH270225 | MH270048 | MH269823 | *A*. sp. 3 RRR 2018 voucher UFPA AA 573 |
| MH270260 | MH270057 | MH269858 | *A*. sp. 3 RRR 2018 voucher UFPA AA 577 |
| MH270226 | MH270049 | MH269824 | *A*. sp. 3 RRR 2018 voucher UFPA AA 580 |
| MH270261 | MH270058 | MH269859 | *A*. sp. 3 RRR 2018 voucher UFPA AA 581 |
| MH270227 | MH270050 | MH269825 | *A*. sp. 3 RRR 2018 voucher UFPA AA 585 |
| MH270228 | MH270051 | MH269826 | *A*. sp. 3 RRR 2018 voucher UFPA AA 586 |
| MH270262 | MH270059 | MH269860 | *A*. sp. 3 RRR 2018 voucher UFPA AA 590 |
| MH270263 | MH270060 | MH269861 | *A*. sp. 3 RRR 2018 voucher UFPA AA 591 |
| MH270264 | MH270061 | MH269862 | *A*. sp. 3 RRR 2018 voucher UFPA AA 592 |
| MH270265 | MH270062 | MH269863 | *A*. sp. 3 RRR 2018 voucher UFPA AA 593 |
| MH270266 | MH270063 | MH269864 | *A*. sp. 3 RRR 2018 voucher UFPA AA 594 |
| MH270267 | MH270064 | MH269865 | *A*. sp. 3 RRR 2018 voucher UFPA AA 595 |
| MH270272 | MH270069 | MH269870 | *A*. sp. 3 RRR 2018 voucher UFPA AA 596 |
| MH270229 | MH270052 | MH269827 | *A*. sp. 3 RRR 2018 voucher UFPA AA 598 |
| MH270230 | MH270053 | MH269828 | *A*. sp. 3 RRR 2018 voucher UFPA AA 599 |
| MH270257 | MH270054 | MH269855 | *A*. sp. 3 RRR 2018 voucher UFPA AA 600 |
| MH270325 | MH270109 | MH269924 | *A*. *teko* voucher AF0900 |
| MH270324 | MH270108 | MH269923 | *A*. *teko* voucher INPA H 36598 |
| MH270323 | MH270107 | MH269922 | *A*. *teko* voucher MNHN 2015.152 |
| MH270209 | MH270007 | MH269807 | *A*. *vote* voucher INPA H 35541 |
| MH270210 | MH270008 | MH269808 | *A*. *vote* voucher INPA H 35542 |
| MH270211 | MH270009 | MH269809 | *A*. *vote* voucher INPA H 35543 |
| MH270212 | MH270010 | MH269810 | *A*. *vote* voucher INPA H 35544 |
| MH270275 | MH270071 | MH269873 | *A*. *xinguensis* voucher INPA H 35471 |
| MH270274 | MH270070 | MH269872 | *A*. *xinguensis* voucher INPA H 35472 |
| MH270279 | MH270075 | MH269877 | *A*. *xinguensis* voucher INPA H 35473 |
| MH270280 | MH270076 | MH269878 | *A*. *xinguensis* voucher INPA H 35474 |
| MH270282 | MH270078 | MH269880 | *A*. *xinguensis* voucher INPA H 35483 |
| MH270277 | MH270073 | MH269875 | *A*. *xinguensis* voucher INPA H 35484 |
| MH270278 | MH270074 | MH269876 | *A*. *xinguensis* voucher INPA H 35485 |
| MH270283 | MH270079 | MH269881 | *A.* *xinguensis* voucher INPA H 35486 |
| MH270276 | MH270072 | MH269874 | *A.* *xinguensis* voucher INPA H 35493 |
| MH270281 | MH270077 | MH269879 | *A.* *xinguensis* voucher INPA-H 35490 |
| NC_037378 | NC_037378 | NC_037378 | *Melanophryniscus moreirae* |
