# Supplementary material for: A new tiny toad species of Amazophrynella (Anura: Bufonidae) from east of the Guiana Shield in Amazonia, Brazil
Source: PeerJ. 2020 Sep 18;8:e9887. doi: 10.7717/peerj.9887 (PMC7505081; doi:10.7717/peerj.9887)
Supplement: Supplemental Information 5 — Data in bold are mean intraspecific divergences. N/C. Not Calculated [file peerj-08-9887-s005.docx]

**Table S2.** Uncorrected p-distances for COI gene of the new species and 16 other *Amazophrynella* species (or candidate species) taken from GenBank (see Document S1). Data in bold are mean intraspecific divergences. N/C. Not Calculated.

|  |  | 1 | 2 | 3 | 4 | 5 | 6 | 7 | 8 | 9 | 10 | 11 | 12 | 13 | 14 | 15 | 16 | 17 | 18 |
| --- | --- | --- | --- | --- | --- | --- | --- | --- | --- | --- | --- | --- | --- | --- | --- | --- | --- | --- | --- |
| 1 | ***A. gardai*** sp. nov. | **0.0234** |  |  |  |  |  |  |  |  |  |  |  |  |  |  |  |  |  |
| 2 | *A. manaos* | 0.0875 | **0.0000** |  |  |  |  |  |  |  |  |  |  |  |  |  |  |  |  |
| 3 | *A.* sp.1 | 0.0875 | 0.0000 | **0.0000** |  |  |  |  |  |  |  |  |  |  |  |  |  |  |  |
| 4 | *A.* *teko* | 0.0875 | 0.0000 | 0.0000 | **0.0000** |  |  |  |  |  |  |  |  |  |  |  |  |  |  |
| 5 | *A.* aff. *vote* sp.1 | 0.1387 | 0.1357 | 0.1357 | 0.1357 | **0.0000** |  |  |  |  |  |  |  |  |  |  |  |  |  |
| 6 | *A.* *vote* | 0.1387 | 0.1357 | 0.1357 | 0.1357 | 0.0000 | **0.0000** |  |  |  |  |  |  |  |  |  |  |  |  |
| 7 | *A*. *vote* sp.2 | 0.1387 | 0.1357 | 0.1357 | 0.1357 | 0.0000 | 0.0000 | **0.0000** |  |  |  |  |  |  |  |  |  |  |  |
| 8 | *A. bokermanni* | 0.1402 | 0.1413 | 0.1413 | 0.1413 | 0.0386 | 0.0386 | 0.0386 | **0.0493** |  |  |  |  |  |  |  |  |  |  |
| 9 | *A.* sp.2 | 0.1454 | 0.1290 | 0.1290 | 0.1290 | 0.0667 | 0.0667 | 0.0667 | 0.0770 | **0.0095** |  |  |  |  |  |  |  |  |  |
| 10 | *A.* sp.3 | 0.1458 | 0.1286 | 0.1286 | 0.1286 | 0.0714 | 0.0714 | 0.0714 | 0.0798 | 0.0048 | **0.0000** |  |  |  |  |  |  |  |  |
| 11 | *A. xinguensis* | 0.1458 | 0.1286 | 0.1286 | 0.1286 | 0.0714 | 0.0714 | 0.0714 | 0.0798 | 0.0048 | 0.0000 | **N/C** |  |  |  |  |  |  |  |
| 12 | *A.* aff. *minuta* sp.1 | 0.1760 | 0.1838 | 0.1838 | 0.1838 | 0.1766 | 0.1766 | 0.1766 | 0.1805 | 0.1855 | 0.1862 | 0.1862 | **0.0000** |  |  |  |  |  |  |
| 13 | *A. minuta* | 0.1760 | 0.1838 | 0.1838 | 0.1838 | 0.1766 | 0.1766 | 0.1766 | 0.1805 | 0.1855 | 0.1862 | 0.1862 | 0.0000 | **0.0000** |  |  |  |  |  |
| 14 | *A. amazonicola* | 0.1911 | 0.1952 | 0.1952 | 0.1952 | 0.1833 | 0.1833 | 0.1833 | 0.1798 | 0.1744 | 0.1738 | 0.1738 | 0.1480 | 0.1480 | **0.0000** |  |  |  |  |
| 15 | *A. siona* | 0.1911 | 0.1952 | 0.1952 | 0.1952 | 0.1833 | 0.1833 | 0.1833 | 0.1798 | 0.1744 | 0.1738 | 0.1738 | 0.1480 | 0.1480 | 0.0000 | **0.0000** |  |  |  |
| 16 | *A. matses* | 0.1911 | 0.1952 | 0.1952 | 0.1952 | 0.1833 | 0.1833 | 0.1833 | 0.1798 | 0.1744 | 0.1738 | 0.1738 | 0.1480 | 0.1480 | 0.0000 | 0.0000 | **0.0000** |  |  |
| 17 | *A. moisesii* | 0.1927 | 0.1952 | 0.1952 | 0.1952 | 0.1849 | 0.1849 | 0.1849 | 0.1814 | 0.1760 | 0.1754 | 0.1754 | 0.1512 | 0.1512 | 0.0032 | 0.0032 | 0.0032 | **0.0032** |  |
| 18 | *M. moreirae* | 0.2113 | 0.2214 | 0.2214 | 0.2214 | 0.2119 | 0.2119 | 0.2119 | 0.2130 | 0.2275 | 0.2286 | 0.2286 | 0.2196 | 0.2196 | 0.2238 | 0.2238 | 0.2238 | 0.2254 | **N/C** |
