# Supplementary material for: A new tiny toad species of Amazophrynella (Anura: Bufonidae) from east of the Guiana Shield in Amazonia, Brazil
Source: PeerJ. 2020 Sep 18;8:e9887. doi: 10.7717/peerj.9887 (PMC7505081; doi:10.7717/peerj.9887)
Supplement: Supplemental Information 6 — Data in bold are mean intraspecific divergences. N/C. Not Calculated [file peerj-08-9887-s006.docx]

**Table S3.** Uncorrected p-distances for 16S gene of the new species and 17 other *Amazophrynella* species (or candidate species) taken from GenBank (see Table S1). Data in bold are mean intraspecific divergences. N/C. Not Calculated.

|  |  | 1 | 2 | 3 | 4 | 5 | 6 | 7 | 8 | 9 | 10 | 11 | 12 | 13 | 14 | 15 | 16 | 17 | 18 | 19 |
| --- | --- | --- | --- | --- | --- | --- | --- | --- | --- | --- | --- | --- | --- | --- | --- | --- | --- | --- | --- | --- |
| 1 | ***A. gardai*** sp. nov. | **N/C** |  |  |  |  |  |  |  |  |  |  |  |  |  |  |  |  |  |  |
| 2 | *A. manaos* | 0.0562 | **0.0079** |  |  |  |  |  |  |  |  |  |  |  |  |  |  |  |  |  |
| 3 | *A.* *teko* | 0.0762 | 0.0501 | **0.0053** |  |  |  |  |  |  |  |  |  |  |  |  |  |  |  |  |
| 4 | *A.* *bilinguis* | 0.0766 | 0.0733 | 0.0735 | **0.0000** |  |  |  |  |  |  |  |  |  |  |  |  |  |  |  |
| 5 | *A.* sp.1 | 0.0814 | 0.0540 | 0.0122 | 0.0822 | **0.0019** |  |  |  |  |  |  |  |  |  |  |  |  |  |  |
| 6 | *A. bokermanni* | 0.1014 | 0.0627 | 0.0795 | 0.0617 | 0.0868 | **N/C** |  |  |  |  |  |  |  |  |  |  |  |  |  |
| 7 | *A.* sp.3 | 0.1091 | 0.0800 | 0.0849 | 0.0285 | 0.0914 | 0.0662 | **0.0206** |  |  |  |  |  |  |  |  |  |  |  |  |
| 8 | *A.* aff. *vote* sp.1 | 0.1098 | 0.0810 | 0.0884 | 0.0569 | 0.0916 | 0.0812 | 0.0698 | **0.0169** |  |  |  |  |  |  |  |  |  |  |  |
| 9 | *A.* *vote* | 0.1135 | 0.0837 | 0.0876 | 0.0511 | 0.0940 | 0.0830 | 0.0651 | 0.0275 | **0.0000** |  |  |  |  |  |  |  |  |  |  |
| 10 | *A.* aff. *vote* sp.2 | 0.1135 | 0.0868 | 0.0810 | 0.0661 | 0.0808 | 0.0870 | 0.0732 | 0.0214 | 0.0337 | **0.0000** |  |  |  |  |  |  |  |  |  |
| 11 | *A.* sp.2 | 0.1155 | 0.0858 | 0.0876 | 0.0340 | 0.0949 | 0.0593 | 0.0296 | 0.0725 | 0.0672 | 0.0752 | **0.0000** |  |  |  |  |  |  |  |  |
| 12 | *A. xinguensis* | 0.1227 | 0.0940 | 0.0928 | 0.0468 | 0.0999 | 0.0668 | 0.0557 | 0.0797 | 0.0672 | 0.0863 | 0.0411 | **0.0046** |  |  |  |  |  |  |  |
| 13 | *A*. *matses* | 0.1334 | 0.0990 | 0.1083 | 0.1024 | 0.1085 | 0.1225 | 0.1112 | 0.1134 | 0.1228 | 0.1168 | 0.1129 | 0.1319 | **0.0004** |  |  |  |  |  |  |
| 14 | *A. amazonicola* | 0.1355 | 0.1130 | 0.1112 | 0.1095 | 0.1136 | 0.1234 | 0.1161 | 0.1175 | 0.1183 | 0.1210 | 0.1159 | 0.1241 | 0.0837 | **0.0216** |  |  |  |  |  |
| 15 | *A.* aff. *minuta* sp.1 | 0.1385 | 0.1063 | 0.1054 | 0.0830 | 0.1096 | 0.1118 | 0.0940 | 0.1040 | 0.1061 | 0.1061 | 0.0995 | 0.1067 | 0.0752 | 0.0771 | **0.0042** |  |  |  |  |
| 16 | *A. siona* | 0.1459 | 0.1160 | 0.1133 | 0.0927 | 0.1151 | 0.1154 | 0.1011 | 0.1111 | 0.1069 | 0.1101 | 0.1014 | 0.1030 | 0.0912 | 0.0641 | 0.0456 | **0.0147** |  |  |  |
| 17 | *A. minuta* | 0.1485 | 0.1165 | 0.1147 | 0.0894 | 0.1189 | 0.1204 | 0.0985 | 0.1111 | 0.1134 | 0.1127 | 0.1048 | 0.1122 | 0.0812 | 0.0863 | 0.0184 | 0.0556 | **0.0223** |  |  |
| 18 | *A. moisesii* | 0.1516 | 0.1171 | 0.1192 | 0.0917 | 0.1206 | 0.1166 | 0.0979 | 0.1042 | 0.0931 | 0.1010 | 0.0990 | 0.1105 | 0.0833 | 0.0849 | 0.0672 | 0.0624 | 0.0732 | **0.0000** |  |
| 19 | *M. moreirae* | 0.1882 | 0.1696 | 0.1756 | 0.1368 | 0.1830 | 0.1624 | 0.1643 | 0.1643 | 0.1706 | 0.1667 | 0.1687 | 0.1746 | 0.1824 | 0.1877 | 0.1637 | 0.1666 | 0.1684 | 0.1797 | **N/C** |
